# Effect of Electroacupuncture vs Sham Treatment on Change in Pain Severity Among Adults With Chronic Low Back Pain: A Randomized Clinical Trial

**DOI:** 10.1001/jamanetworkopen.2020.22787

**Published:** 2020-10-27

**Authors:** Jiang-Ti Kong, Chelcie Puetz, Lu Tian, Isaac Haynes, Eunyoung Lee, Randall S. Stafford, Rachel Manber, Sean Mackey

**Affiliations:** 1 Division of Pain Medicine, Department of Anesthesiology, Perioperative and Pain Medicine, Stanford University School of Medicine, Stanford, California; 2 Department of Biomedical Data Science, Stanford University School of Medicine, Stanford, California; 3 Department of Medicine, Stanford University School of Medicine, Stanford, California; 4 Department of Psychiatry, Stanford University School of Medicine, Stanford, California

## Abstract

**Importance:**

Chronic low back pain has high societal and personal impact but remains challenging to treat. Electroacupuncture has demonstrated superior analgesia compared with placebo in animal studies but has not been extensively studied in human chronic pain conditions.

**Objective:**

To evaluate the treatment effect of real electroacupuncture vs placebo in pain and disability among adults with chronic low back pain and to explore psychophysical, affective, and demographic factors associated with response to electroacupuncture vs placebo in treating chronic low back pain.

**Design, Setting, and Participants:**

This double-blind randomized clinical trial was conducted between August 2, 2016, and December 18, 2018, at a single center in Stanford, California. Primary outcomes were collected at approximately 2 weeks before and after intervention. Participants included English-speaking adults with at least 6 months of chronic low back pain, pain intensity of at least 4 on a scale of 0 to 10, and no radiculopathy. Data analyses for this intent-to-treat study were conducted from June 2019 to June 2020.

**Interventions:**

Twelve sessions of real or placebo (sham) electroacupuncture administered twice a week over 6 weeks.

**Main Outcomes and Measures:**

The main outcome was change in pain severity from baseline to 2 weeks after completion of treatment, measured by the National Institutes of Health PROMIS pain intensity scale. A secondary outcome was change in the Roland Morris Disability Questionnaire (RMDQ). Baseline factors potentially associated with these outcomes included psychophysical testing (ie, thermal temporal summation, conditioned pain modulation, pressure pain threshold), participant’s self-report (ie, widespread pain, coping strategies, expectations, self-efficacy, and pain catastrophizing), and demographic characteristics (eg, age, sex, and race).

**Results:**

A total of 121 adults were recruited to the study, among whom 59 participants (mean [SD] age, 46.8 [11.9] years; 36 [61.0%] women) were randomized to real electroacupuncture and 62 participants (mean [SD] age, 45.6 [12.8] years; 33 [53.2%] women) were randomized to sham electroacupuncture. At baseline, the mean (SD) PROMIS T-score was 50.49 (3.36) in the real electroacupuncture group and 51.71 (4.70) in the sham acupuncture group, and the mean (SD) RMDQ score was 10.16 (4.76) in the real electroacupuncture group and 10.03 (5.45) in the sham acupuncture group. After adjusting for baseline pain scores, there was no statistically significant difference between groups in change in T-scores 2 weeks after completion of treatment (real electroacupuncture: −4.33; 95% CI, −6.36 to −2.30; sham acupuncture: −2.90; 95% CI, −4.85 to −0.95; difference: −2.09; 95% CI, −4.27 to 0.09; *P* = .06). After adjusting for baseline RMDQ, there was a significantly greater reduction in RMDQ in the real electroacupuncture group (−2.77; 95% CI, −4.11 to −1.43) compared with the sham electroacupuncture group (−0.67; 95% CI, −1.88 to 0.55; difference: −2.11; 95% CI, −3.75 to −0.47; *P* = .01). Within the real electroacupuncture group, effective coping at baseline was associated with greater RMDQ reduction (*r* = −0.32; 95% CI, −0.54 to −0.05; *P* = .02), and White race was associated with worse outcomes in PROMIS score (β = 3.791; 95% CI, 0.616 to 6.965; *P* = .02) and RMDQ (β = 2.878; 95% CI, 0.506 to 5.250; *P* = .02).

**Conclusions and Relevance:**

This randomized clinical trial found no statistically significant difference in change in PROMIS pain score in real electroacupuncture vs sham electroacupuncture. There was a statistically significant treatment effect for the secondary outcome of RMDQ compared with sham electroacupuncture. Effective coping skills and non-White race were associated with response to electroacupuncture.

**Trial Registration:**

ClinicalTrials.gov Identifier: NCT02890810

## Key Points

**Question:**

What is the effect of electroacupuncture vs sham treatment on pain severity in adults with chronic low back pain?

**Findings:**

In this randomized clinical trial including 121 adults with chronic low back pain, there was no significant difference between real and sham electroacupuncture conditions in reduction in pain scores 2 weeks after treatment; however, real electroacupuncture resulted in statistically and clinically greater reduction in back pain–specific disability compared with the sham control. Having coping strategies and not being White race were associated with treatment response to real electroacupuncture.

**Meaning:**

This randomized clinical trial found no difference in pain reduction between real and sham electroacupuncture, but there was greater reduction in back pain–specific disability compared with sham control.

## Teaser

This randomized clinical trial examines real vs sham electropuncture in treating chronic low back pain in adults.

## Introduction

Chronic low back pain is a leading cause of disability in the United States, second only to ischemic heart disease and lung cancer.^1^ Pharmacological and surgical treatments for chronic low back pain are associated with adverse effects, such as addiction and surgical complications.^2,3^ Most clinical trials on acupuncture for chronic low back pain thus far involve manual acupuncture and found similar magnitudes of effect between real and sham (placebo) acupuncture.^4,5,6^ Preclinical studies suggest that electroacupuncture may lead to stronger analgesic outcomes than manual acupuncture.^7,8^ However, the few controlled clinical trials that evaluated electroacupuncture in chronic low back pain had methodological limitations, such as absence of blinding and small sample sizes,^9,10^ and have not examined factors associated with response.

It is important to identify factors associated with clinical response to acupuncture, because, although recent evidence established acupuncture as an effective treatment of chronic low back pain,^11,12^ the response rate to acupuncture is approximately 40% to 60%.^5,6^ As the US and other health care systems begin to rely on acupuncture as a mainstay of opioid-sparing therapy for chronic low back pain,^13,14^ early identification of responders would improve triaging and facilitate the pragmatic implementation of acupuncture. The existing knowledge on factors associated with clinical response to acupuncture is from research on manual acupuncture, mainly focused on baseline pain and function, demographic characteristics, and expectations of benefits, with limited overall predictive power.^15,16,17^

This randomized clinical trial investigated the effectiveness of electroacupuncture in treating chronic low back pain, but with the additional aim of identifying factors associated with treatment response using change in pain reduction and disability as clinical outcomes. The association between prespecified key factors associated with response and pain intensity formed the basis of our power calculation. Consistent with our aim to identify differential factors associated with response to electroacupuncture, we used the least active yet credible sham control (nonpenetrating Streitberger needles^18^ on off-meridian points without active stimulation)^19^ to better differentiate the real from the sham electroacupuncture models. To maximize generalizability to clinical practice, we administered the interventions at the clinics of multiple community acupuncturists near participants’ residences using a semistandardized treatment protocol.^20^

We examined a broad range of potential factors associated with clinical response. In addition to those previously examined, we evaluated impairment in central pain regulation as potential associated factors. We specifically focused on augmented ascending facilitation and reduced descending inhibition, which are considered key mechanisms in the development and maintenance of chronic pain^21,22^ and can be approximated by specific measures from quantitative sensory testing.^23,24^ We also examined psychological factors associated with chronic pain, including pain catastrophizing,^25^ self-efficacy in managing pain,^26^ and coping.^27^

## Methods

### Study Design and Participants

The Trial Protocol in Supplement 1 was reviewed and approved by the Stanford University institutional review board, and written informed consent was obtained from all participants before any experimental procedures. The reporting of the study follows the Consolidated Standards of Reporting Trials (CONSORT) reporting guideline.

We conducted a single-center, parallel-arm, randomized clinical trial comparing real vs sham electroacupuncture in treating patients with chronic low back pain. The main inclusion criteria were adults aged 21 to 65 years who were fluent in English, had chronic low back pain for at least 6 months (as defined by the National Institutes of Health Task Force on Research Standards for Chronic Low Back Pain^28^), and had back pain intensity of at least 4 on a 0 to 10 numerical rating scale in the past month. The main exclusion criteria were radicular pain due to disc compression or spinal stenosis evidenced by examination or magnetic resonance imaging, other pain conditions with an intensity greater than the low back pain, or recent acupuncture experience within the last 3 years (Figure). Additional eligibility details are listed in eAppendix 1 in Supplement 2.

**Figure.**
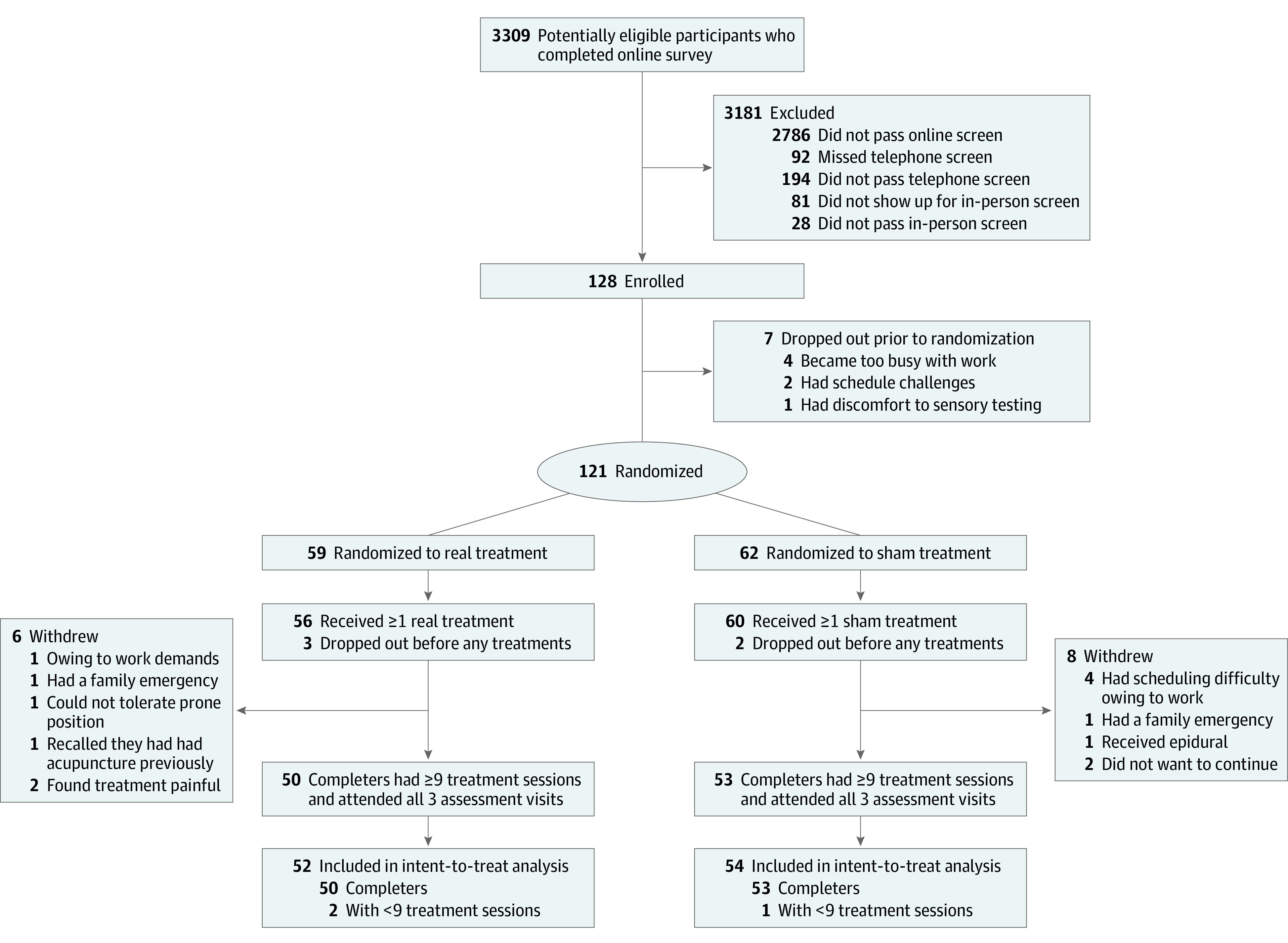
Participant Recruitment Flowchart

### Interventions

The real and sham treatments included twelve 45-minute sessions over 6 weeks. In both interventions, the participant was positioned comfortably in the prone position and received approximately 30 minutes of active or sham electrical stimulation after all needles were placed.^20^ The detailed treatment protocol is provided in the Trial Protocol in Supplement 1.

Participants were randomized with equal chances to receive real or sham treatments, stratified to 10 acupuncturists. A participant first selected an acupuncturist based on geographic preference and was then randomized to treatment arm by a person unrelated to the study, using the randomization table corresponding to the patient’s selected acupuncturist. Both the participant and the outcome assessor, but not the acupuncturist, were blinded to treatment assignment.

### Outcome

#### Primary Clinical Outcome

The intensity of back pain was measured by the National Institutes of Health PROMIS pain intensity instrument, using T-scores calibrated to the general US population.^29^ The primary outcome was the change in T-score (posttreatment score – pretreatment score).

#### Secondary Clinical Outcome

Back pain–specific disability was measured by the Roland Morris Disability Questionnaire (RMDQ).^30^ The secondary outcome was the change in the total RMDQ score (posttreatement score – pretreatment score). The scoring of the RMDQ ranges from 0 to 24, in which 0 indicates no disability and 24, highest level of back-related disability.

#### Factors Associated With Response

The 14 prespecified potential factors associated with response with details of their measurement^24,31,32,33,34,35,36,37,38,39,40,41,42^ are presented in Table 1. Both PROMIS pain and RMDQ scores were collected at the pretreatment and posttreatment visits, approximately 2 weeks before the first treatment session and 2 weeks after the last treatment session. All variables were collected at the pretreatment visit.

**Table 1.** Summary of 14 Prespecified Factors Associated With Response

Factor	Measurement details	Range	Hypothesized influence
Sex	1 = Women, 0 = Men	0-1	Women would have better response to all treatments
Race	1 = White, 0 = Other	0-1	White individuals would respond more to all treatments
Age	Continuous variable	21-65 y	Younger individuals would respond better to all treatments
Widespread pain	Measured as the total number of areas marked by the participant as painful on a standardized digital body map.	0-72	Greater value would be associated with greater response to verum treatment
Pressure pain threshold	Measured as the mean of the pain threshold to blunt pressure at the top of the trapezius muscle bilaterally, delivered by an algometer (FDK20; Wagner Instruments)^24^	0-12 (kg/cm^2^)	Lower pressure pain threshold would be associated with greater response to sham EA (primary hypothesis)^32^
TS magnitude	Measured by the difference in the pain ratings to the first and to the most painful pulse in a train of 10 identical noxious heat pulses.^33^ These pulses were delivered to the thenar eminence via a contact heat thermode (Medoc Pathway), with individualized stimulating temperatures^34^	0-100 (Visual analog scale)	Greater magnitude of TS would be associated with greater response to verum EA (primary hypothesis)^35^
TS			
Baseline	Measured at baseline^34^	33-44 °C	No specific hypothesis
Peak	Highest temperature measured^34^	44-51 °C	No specific hypothesis
Conditioned pain modulation	Measured by the decrease in the pain rating on a visual analog scale of a testing stimulus, temporal summation magnitude at the thenar eminence, resulting from the application of a noxious conditioning stimulus,^36^ submersion of the contralateral foot in a 10 °C cold bath for 2 min.^34^ The TS paradigm was performed immediately before and during the last 30 s of the cold bath, and conditioned pain modulation was the difference in the TS performed at these times	0-100	Lower score would be associated with greater response to real electroacupunture^37^
Positive expectation	Measured as the sum of the 3 positive expectation questions on the SETS^38^	3-21	Greater positive expectation would be associated with greater response to all treatments^39^
Negative expectation	Measured as the sum of the 3 negative expectation questions from SETS^38^	3-21	Greater negative expectation would be associated with lower response to all treatments
Pain self-efficacy	Measured as the sum of PSEQ^40^ items	50-500	Greater self-efficacy in managing pain would be associated with greater response to all treatments^31^
Coping strategies	Measured as the sum of 21 items from the CSQ,^41^ after subtracting the 6 items on catastrophizing (the only negative items). These 21 items assess strategies, including distraction, ignoring pain, distancing, coping self-statements, and praying	0-126	Greater CSQ would be associated with greater response to all treatments^31^
PCS	The sum of the 13 items from the pain catastrophizing scale.^42^ The PCS has components in rumination, magnification and feeling of helplessness	0-52	Greater PCS would be associated with less response to all treatments^31^

Abbreviations: CSQ, Coping Strategy Questionnaire; EA, electroacupuncture; PCS, Pain Catastrophizing Scale; PSEQ, Pain Self-efficacy Questionnaire; SETS, Stanford Expectations of Treatment Scale; TS, Temporal summation.

### Study Power

We powered the study to detect associations (correlations) between key baseline factors (eg, temporal summation [TS]) and the primary clinical outcome (ie, pain reduction). We first computed the number of participants needed to find an association between TS and pain reduction within the real electroacupuncture arm. We estimated that, with 80% power and .05 two-tailed α, 50 participants would be needed to detect a correlation of approximately 0.4, which represents a moderate but clinically meaningful association. This correlation is achievable based on previous findings of associations between pain reduction and TS,^43^ and separately, pressure pain threshold.^44^ Second, we assessed if the association is stronger in the real arm than the sham arm. Fifty patients per group would provide 80% power (with α = .05) to detect a difference between a correlation of 0.28 in the real condition and a correction of −0.28 in the sham condition. We would need approximately 60 participants per arm assuming 20% attrition.

### Statistical Analysis

#### Treatment Effect Estimation and Blinding Assessment

We used analysis of covariance to estimate the effect of the treatment interventions on pain and RMDQ, adjusted to the baseline pain or RMDQ. We assessed the success of blinding at the end of the 12th treatment (Trial Protocol in Supplement 1) and its influence on treatment effect. The success of blinding was quantified by Bang blinding index within each arm.^45,46^ We then assessed the impact of blinding on the treatment effect by including a blinded variable (0 if the participant correctly guessed their treatment group and 1 otherwise) in the analysis of covariance.

#### Identification of Factors Associated With Response

We explored factors associated with treatment response via 3 steps, whereby the first 2 steps were used to reduce the number of factors in the multivariable models in the third step. First, we examined the associations between each factor and the 2 main outcomes separately within the real and sham conditions. For continuous variables, we estimated the partial correlation coefficients, and for the categorical variables (ie, sex and race) we tested linear regression models, adjusted for baseline pain and RMDQ scores. Second, we quantified the interaction between each factor and treatment allocation in linear regression models. Each model contained 4 covariates: baseline dependent variable, binary treatment indicator, factor of interest, and interaction between the factor of interest and treatment. We accounted for multiple testing by applying the Benjamin-Hotchberg procedure to control the family-wise type 1 error rate at the α = .05 level. Third, the factors of interest for the models were selected based on a cutoff *P* value of .05 from the first step to build models to estimate change in pain and RMDQ. Additionally, for the analyses based on the full sample, a treatment interaction term with the lowest *P* value from the second step was included.

#### Missing Values

The missingness of our data was examined by Little missing completely at random test^47^ from the BaylorEdPsych package in R statistical software version 3.5.2 (R Project for Statistical Computing). The *P* value for the Little missing completely at random test was .30, not suggesting any violation of the missing completely at random assumption. We thus performed complete-case analysis on our primary outcomes (ie, pain and RMDQ scores) according to the intention-to-treat (ITT) principle.^48^ Completion cases were defined as individuals who presented for both the pretreatment and posttreatment visits regardless of the number of treatment sessions they received.

#### Outlier and Sensitivity Analysis

Sensitivity analysis (eAppendix 2 in Supplement 2) identified a single outlier from the real electroacupuncture condition with change in pain score of more than 2 interquartile ranges above the third quartile^49^ and was singularly responsible for the 2 univariate associations involving change in pain. We therefore conducted analyses with and without this outlier.

## Results

### Participant Characteristics

A total of 121 participants were recruited. Of 59 individuals randomized to the real electroacupuncture condition (mean [SD] age, 46.8 [11.9] years; 36 [61.0%] women), 52 attended the assessment visits and were included in the ITT analysis (50 participants had ≥9 treatment sessions). Of 62 individuals randomized to the sham condition (mean [SD] age, 45.6 [12.8] years; 33 [53.2%] women), 54 attended the assessment visits and were included in the ITT analysis (53 participants had ≥9 treatment sessions). The baseline characteristics were comparable between conditions (eTable 1 in Supplement 2). At baseline, the mean (SD) T-score was 50.49 (3.36) in the real electroacupuncture condition and 51.71 (4.79) in the sham acupuncture condition. The mean (SD) RMDQ score was 10.16 (4.76) in the real electroacupuncture group and 10.03 (5.45) in the sham electroacupuncture group.

### Treatment Effect

#### Pain T-Score and RMDQ

At the final assessment 2 weeks after completion of treatment, there was no statistically significant difference between groups in change in pain T-scores (real electroacupuncture: −4.33; 95% CI, −6.36 to −2.30; sham acupuncture: −2.90; 95% CI, −4.85 to −0.95; unadjusted difference: −1.50; 95% CI, −3.72 to 0.72; *P* = .18; adjusted difference: −2.09; 95% CI, −4.27 to 0.09; *P* = .06). However, there was a significant difference in the reduction of RMDQ scores between groups, with a greater reduction in the real electroacupuncture group (real electroacupuncture: −2.77; 95% CI, −4.11 to −1.43; sham electroacupuncture: −0.67; 95% CI, −1.88 to 0.55; unadjusted difference: −2.10; 95% CI, −3.89 to −0.31; *P* = .02), and the difference was unaffected by adjustment to the baseline RMDQ levels (adjusted difference: −2.11; 95% CI, −3.75 to −0.47; *P* = .01).

#### Blinding Assessment, Outlier, and Impact on Treatment Effect 

Of 48 individuals assigned to the real electroacupuncture condition who provided data on blinding, 28 (58.3%) correctly guessed their treatment. Of 46 individuals assigned to the sham condition who provided data on blinding, 20 (42.5%) correctly guessed their assignment. The Bang blinding index was 0.50 (95% CI, 0.32 to 0.68) for the real electroacupuncture group and 0.20 (95% CI, −0.07 to 0.46) for the sham group. These indices suggest that the blinding for the sham condition was adequate while that for the real electroacupuncture condition was not (eTable 2 in Supplement 2). After accounting for the blinding status of each participant, the treatment effect on change in RMDQ remained significant (β = −2.23; 95% CI, −4.03 to −0.42; *P* = .02), while the treatment effect on change in PROMIS pain score remained not statistically significant (β = −2.09; 95% CI, −4.56 to 0.37; *P* = .10).

After removal of the single outlier, the treatment effect on both changes in PROMIS pain and RMDQ scores became significant (change in pain: β = −2.48, 95% CI, −4.51, −0.46; *P* = .02; change in RMDQ: β = −2.13; 95% CI, −3.79 to −0.48; *P* = .01). These treatment effects remained significant after accounting for blinding status (change in pain: β = −2.61; 95% CI, −4.90 to −0.33; *P* = .03; change in RMDQ: β = −2.24; 95% CI, −4.07 to −0.41; *P* = .02).

### Analysis of Factors Associated With Treatment Response

Results from the univariate analysis in the real electroacupuncture condition are shown in Table 2. The partial correlation between coping and change in RMDQ remained significant regardless of the outlier, whereas the correlations between change in pain and TS or expectations disappeared on removal of the outlier. White race was associated with worse outcomes in pain with the outlier (β = 4.408; 95% CI, 0.880 to 7.936; *P* = .02) and excluding the outlier (β = 3.791; 95% CI, 0.616 to 6.965; *P* = .02) and in RMDQ (with outlier: β = 2.840; 95% CI, 0.507 to 5.172; *P* = .02; excluding outlier: β = 2.878; 95% CI, 0.506 to 5.250; *P* = .02). No relationships between the factors of interest and the outcomes were observed in the sham condition (eTable 3 in Supplement 2). Furthermore, in a post hoc analysis for the entire sample (excluding the outlier), we found that compared with participants who were not Asian, Asian participants experienced statistically significant, greater reductions in both pain (change, 2.85; 95% CI, 0.13 to 5.58; P = .04) and RMDQ (change, 2.45; 95% CI, 0.02 to 4.89; *P* = .049). None of the prespecified factors of interest demonstrated statistically significant interactions with treatment allocation (eTable 4 in Supplement 2). The results of the models, with or without the outlier, are shown in Table 3 for the real acupunctured condition and Table 4 for both conditions.

**Table 2.** Univariate Analyses Within Real Electroacupuncture Group, With and Without Outlier

Baseline variable	No.^a^	Change in pain				Change in RMDQ			
		All participants (n = 51)^b^		Excluding outlier (n = 50)		All participants (n = 52)		Excluding outlier (n = 51)	
		*r *	*P* value	*r *	*P* value	*r *	*P* value	*r *	*P* value
**Continuous variables**									
Age	52	−0.13	.38	−0.12	.41	0.10	.49	0.10	.49
CSQ score^c^	52	−0.28	.05	−0.20	.17	−0.31	.03	−0.31	.03
Pain self-efficacy^d^	52	−0.01	.96	0.13	.38	0.09	.54	0.10	.50
Pain catastrophizing^e^	52	0.23	.11	0.10	.51	−0.08	.60	−0.08	.52
Mean pressure pain threshold^f^	52	−0.14	.33	−0.14	.32	−0.04	.77	−0.04	.78
Base temperature^g^	51	0.27	.05	0.26	.07	0.18	.21	0.18	.23
Peak temperature^h^	51	0.11	.44	0.13	.36	0.15	.32	0.15	.32
Mean temperature, thenar eminence^i^	51	0.32	.02	0.14	.35	0.11	.43	0.11	.46
Conditioned pain modulation^j^	51	0.02	.88	0.04	.79	0.18	.21	0.18	.21
Widespread pain^k^	52	−0.02	.88	0.05	.74	0.11	.43	0.11	.41
Positive expectations^l^	50	−0.33	.02	0.20	.18	−0.05	.74	−0.05	.80
Negative expectations^m^	50	0.13	.37	0.09	.53	0.07	.63	0.07	.65
**Categorical variables**									
Women	52	4.256 (0.755 to 7.756)^n^	.02	3.528 (0.356 to 6.700)^n^	.03	1.711 (−0.734 to 4.155)^n^	.17	1.766 (−0.744 to 4.277)^n^	.16
White race	52	4.408 (0.880 to 7.936)^n^	.02	3.791 (0.616 to 6.966)^n^	.02	2.840 (0.507 to 5.172)^n^	.02	2.878 (0.506 to 5.250)^n^	.02

Abbreviations: CSQ, Coping Strategy Questionnaire; RMDQ, Roland Morris Disability Questionnaire.

^a^ Indicates the total number of participants who provided values on that variable at baseline.

^b^ One participant did not provide baseline pain level. Therefore 51 out of the 52 completers in the real electroacupuncture condition had change in pain data.

^c^ CSQ: coping as measured by the coping strategy questionnaire.

^d^ Measured by the pain self-efficacy questionnaire.

^e^ Measured by the pain catastrophizing scale.

^f^ Measured as the mean pressure pain threshold measured on bilateral trapezius.

^g^ Measured as the individualized baseline temperature of the heat pulses used to generate temporal summation.

^h^ Measured as the individualized peak temperature of the heat pulses used to general temporal summation.

^i^ Measured as the mean temporal summation measured on both hands at the thenar eminence.

^j^ Measured as the magnitude of conditioned pain modulation.

^k^ Measured as the sum of the total number of body areas marked as in pain by the participant, a measure of widespread pain.

^l^ Measured by summing the 3 positive items from the Stanford Expectation of Treatment scale.

^m^ Measured by summing the 3 negative items from the Stanford Expectation of Treatment scale.

^n^ Data are provided as β (95% CI).

**Table 3.** Multivariable Models Within Real Electroacupuncture Condition, With or Without the Outlier

Outcome	With outlier		Outlier removed	
	β (95% CI)	*P* value	β (95% CI)	*P* value
**Change in pain**				
No.	48		47	
Intercept	32.489 (6.204 to 58.773)	.03	28.473 (2.406 to 54.539)	.03
Baseline pain	−0.704 (−1.209 to −0.200)	.007	−0.656 (−1.151 to −0.160)	.01
Women	3.45 (−0.023 to 6.923)	.05	2.973 (−0.461 to 6.407)	.09
White race	2.046 (−1.593 to 5.684)	.26	2.256 (−1.304 to 5.817)	.21
Mean baseline temporal summation	0.109 (0.024 to 0.193)	.01	0.066 (−0.029 to 0.162)	.17
Positive expectations	−0.604 (−1.111 to −0.097)	.02	−0.400 (−0.947 to 0.146)	.15
Adjusted *R*^2^	0.346	.01	0.213	<.001
**Change in RMDQ**				
No.	49		48	
Intercept	4.304 (−1.060 to 9.668)	.11	4.744 (−0.861 to 10.348)	.10
Baseline RMDQ	−0.444 (−0.681 to −0.207)	<.001	−0.464 (−0.712 to −0.216)	<.001
White race	2.454 (0.133 to 4.774)	.04	2.514 (0.167 to 4.860)	.04
Coping strategies	−0.057 (−0.119 to 0.006)	.08	−0.06 (−0.124 to 0.004)	.07
Adjusted *R*^2^	0.316	<.001	0.307	<.001

Abbreviation: RMDQ, Roland Morris Disability Questionnaire.

**Table 4.** Multivariable Models in the Entire Sample, With or Without the Outlier

Outcome	With outlier		Outlier removed	
	β (95% CI)	*P* value	β (95% CI)	*P* value
**Change in pain**				
No.	97		96	
Intercept	28.294 (14.344 to 42.244)	<.001	25.279 (11.251 to 39.306)	.001
Baseline pain	−0.533 (−0.808 to -0.258)	<.001	−0.485 (−0.759 to -0.211)	.001
Treatment	−6.651 (−10.687 to −2.615)	.002	−5.895 (−9.930 to −1.859)	.005
Women^a^	1.754 (−0.437 to 3.946)	.12	1.496 (−0.673 to 3.665)	.17
White race^b^	1.997 (−0.280 to 4.274)	.09	1.805 (−0.441 to 4.051)	.11
Mean temporal summation^c^	0.085 (0.028 to 0.142)	.004	0.061 (0 to 0.121)	.05
Positive expectation^d^	−0.463 (−0.798 to −0.129)	.007	−0.356 (−0.701 to −0.011)	.04
Pain catastrophizing^e^	−0.155 (−0.320 to 0.009)	.06	−0.147 (−0.309 to 0.015)	.08
Interaction of treatment and pain catastrophizing	0.299 (0.066 to 0.531)	.01	0.232 (−0.006 to 0.469)	.06
Adjusted *R*^2^	0.270	<.001	0.195	<.001
**Change in RMDQ**				
No.	106		105	
Intercept	6.771 (3.132 to 10.411)	<.001	6.891 (3.173 to 10.609)	<.001
Baseline RMDQ	−0.355 (−0.514 to −0.197)	<.001	−0.361 (−0.523 to −0.199)	<.001
Treatment	−3.222 (−5.773 to −0.671)	.01	−3.203 (−5.768 to −0.638)	.02
White race	0.331 (−1.948 to 2.610)	.77	0.350 (−1.942 to 2.642)	.76
CSQ^f^	−0.058 (−0.103 to −0.013)	.01	−0.059 (−0.105 to −0.014)	.01
Interaction of treatment and White race	2.107 (−1.170 to 5.384)	.21	2.128 (−1.166 to 5.422)	.20
Adjusted R^2^	0.260	<.001	0.256	<.001

Abbreviations: CSQ, Coping Strategy Questionnaire; RMDQ, Roland Morris Disability Questionnaire.

### Adverse Effects

No serious adverse effects were reported by any participants in the study. eTable 5 in Supplement 2 provides details on minor adverse effects.

## Discussion

This randomized clinical trial found no significant difference in chronic low back pain scores between real and sham electroacupuncture treatment. Post hoc analyses found a significant treatment effect of electroacupuncture in reducing disability associated with chronic low back pain. Consistent with previous research^15,16,17^ and likely related to regression to mean, participants in the real treatment arm who had greater pain intensity and RMDQ scores experienced greater improvement in RMDQ scores. Unique to this study was the association between effective coping and greater RMDQ reduction and between White race and less reduction in both pain and RMDQ. Finally, after eliminating a single outlier, the treatment effect became significant in RMDQ and pain scores.

### Treatment Effect of Electroacupuncture

As one of the first randomized clinical trials evaluating electroacupuncture in treating chronic low back pain, our study found a treatment effect in RMDQ. This treatment effect (ie, between-arm difference in change in RMDQ) was present with or without the outlier and after accounting for blinding status. This finding was clinically significant, falling within the range of minimally clinically important difference of RMDQ (ie, 2-8 points).^50,51,52^ By comparison, the treatment effects discovered on previous trials was between 0.1 and 1.7 points.^5,53,54^

Consistent with our original intent to maximize the difference between the real and sham electroacupuncture conditions to facilitate differential model-building, we selected a strong real electroacupuncture protocol. It consisted of active electrical stimulation,^7^ more than 20 needles per session,^55^ and biweekly treatment frequency,^56^ all of which are supported by existing studies and likely contributed to the robust treatment effect in RMDQ observed in our study.

The between-arm difference in pain reduction was not statistically significant for the full sample. Existing literature suggests that clinically significant minimally clinically important difference of PROMIS pain instruments are between 2 to 3 points of change in T-score.^57,58^ We note that the between-arm difference in pain reduction became clinically and statistically significant on removal of the single outlier.

Finally, interpretation of our results on treatment effect should be tempered by the fact that our study was originally designed to capture univariate associations between patient characteristics and clinical outcomes in the real acupuncture arm. The treatment effect in RMDQ, although robust, was captured in post hoc analyses. Larger studies are needed to replicate our findings. Post hoc power calculation suggests we would need 72 participants per arm to detect treatment effect in RMDQ and 91 participants per arm to detect treatment effect in pain with 80% power.

### Factors Associated With Treatment Response

Unique to our study is the finding of an association between positive coping strategies and functional improvement, seen both on the univariate and multivariate analyses. In a 2015 study, Bishop et al^31^ found a similar association in an observational cohort of patients with mixed back pain causes. Our study advances the understanding on the role of coping in response to acupuncture by confirming its association with RMDQ reduction in a randomized clinical trial in a homogenous participant sample of individuals with chronic low back pain.

Contrary to our original hypothesis, the association between TS and change in pain was unstable and vanished on removal of a single outlier. This, in contrast to the stable relationship between coping and change in RMDQ, may be explained by the paramount role of the so-called *emotional brain* in the development and maintenance of chronic pain.^59,60^ Psychological variables may be stronger factors associated with response and amplifiers of chronic pain than somatosensory variables, such as those measured by quantitative sensory testing. As such, future studies should continue to investigate cognitive-emotional factors associated with acupuncture response and may consider combining acupuncture with psychological interventions.^61^

Finally, we found that White race was associated with worse outcomes in pain and RMDQ in the real electroacupuncture condition. This racial influence may be driven by differences in cultural backgrounds, in that participants with backgrounds that include traditional Chinese medicine may be more likely to respond to acupuncture.^62^ Our study was conducted in Stanford, California, where most of the racial/ethnic minority population are of East Asian descent and are open to receiving acupuncture and for treatment of various conditions, including chronic pain. Future studies should confirm our results in larger multicultural samples and test the interaction between cultural background and treatment allocation.

### Limitations

Our study has some limitations. First, our study does not quantify the specific effect of electroacupuncture vs manual acupuncture, which could be assessed in a 3-arm study that includes a placebo control, manual acupuncture, and electroacupuncture. Second, whereas, to our knowledge, ours is the first study on acupuncture for chronic low back pain that has assessed and accounted blinding status in analyses, only 48 of 52 participants in the real electroacupuncture condition and 46 of 54 participants in the sham condition provided blinding data at study completion. Although the missing blinding data were due to implementation imperfections and not to participants’ refusal to answer the questions, we cannot rule out the possibility that results may have been different if all participants had provided blinding data. However, our analysis did demonstrate a statistically significant treatment effect on RMDQ after accounting for the available blinding status in every individual. Thirdly, our outcome collection spanned approximately 10 weeks (2 weeks before and 2 weeks after 6 weeks of interventions). We designed our study in this manner to capture the largest possible treatment effect assuming most benefits emerge immediately after our interventions. Longer outcomes are more meaningful clinically and should be collected and analyzed in future studies.

## Conclusions

This randomized clinical trial found no significant difference in change in pain scores between real and sham electroacupuncture. However, to our knowledge, this is the first study to demonstrate a statistically and clinically significant treatment effect of electroacupuncture on disability associated with chronic low back pain in a randomized clinical trial. Our study also contributes to current knowledge on the patient factors associated with clinical response to electroacupuncture treatment. If validated, these findings may help match people to treatment. For example, low scores on the coping strategies questionnaire could identify individuals who may need psychological intervention alone or as an augmentation to electroacupuncture.

## Notes

Supplement 1. **Trial Protocol**

Supplement 2. **eAppendix 1.** Full Inclusion and Exclusion Criteria **eTable 1.** Participant Baseline Characteristics **eTable 2.** Blinding Analysis **eTable 3.** Univariate Analysis Within the Sham Arm **eTable 4.** Treatment Heterogeneity Analysis **eAppendix 2.** Sensitivity Analysis **eTable 5.** Summary on Type and Frequency of Adverse Events

Supplement 3. **Data Sharing Statement**
