# Error in Conflict of Interest

**DOI:** 10.1001/jamanetworkopen.2022.9687

**Published:** 2022-04-06

In the Original Investigation titled “Effect of Electroacupuncture vs Sham Treatment on Change in Pain Severity Among Adults with Chronic Low Back Pain: A Randomized Clinical Trial”^1^ published October 27, 2020, the conflict of interest disclosure statement should have read as follows: “Dr Mackey reported receiving grants from National Center for Complementary and Integrative Medicine during the conduct of the study; serving as president of the American Academy of Pain Medicine from 2014-2015, as a member of the American Society of Anesthesiologists Pain Committee from 2008-2019, and as a member of the advisory board of the American Chronic Pain Association (a nonprofit organization focused on patient education about pain), and reported receiving no compensation from these entities; and also reported receiving travel reimbursement from the American Academy of Pain Medicine, Tarsus Group, Neurovations, International Neuromodulation Society, and FDA-ACCTION to present pain research findings.” This article has been corrected.^1^
