# Supplementary material for: Effect of Electroacupuncture vs Sham Treatment on Change in Pain Severity Among Adults With Chronic Low Back Pain: A Randomized Clinical Trial
Source: JAMA Netw Open. 2020 Oct 27;3(10):e2022787. doi: 10.1001/jamanetworkopen.2020.22787 (PMC7592030; doi:10.1001/jamanetworkopen.2020.22787)
Supplement: Supplement 1. — Trial Protocol [file jamanetwopen-e2022787-s001.pdf]

Clinical Intervention Project Protocol:

Predicting Analgesic Response to Acupuncture – A Practical Approach

Predicting Analgesic Response to Acupuncture:

A randomized, placebo-controlled, subject and assessor blinded, 100-subject Clinical Trial of Electro-Acupuncture In the Treatment of Chronic Low Back Pain

Study Chairman or Principal Investigator:

Jiang-Ti Kong, MD, Instructor, Department of Anesthesiology – Stanford University School of Medicine

Supported by:
The National Center for Complementary and Integrative Health

K23 AT008477

Study Intervention Provided by:

N/A

Tool Revision History

Version Number: 1.0

Version Date: Dec 8, 2015

Summary of Revisions Made:

Version Number: 2.0

Version Date: 2/15/2016

Summary of Revisions Made:

1. Addition of acupuncturists to the program
2. Clarification of study timeline
3. Revision and clarification of inclusion and exclusion criteria
4. Revision of treatment protocol to clarify difference between current protocol and P01 Project 3
5. Revision of study consents to clarify difference between current protocol and P01 Project 3
6. Clarification on procedures of trial monitoring

Version Number: 3.0

Version Date: 05/18/2016

Summary of Revisions Made:

Personnel

1. Revision of **protocol personnel** (full list of acupuncturists and research coordinator)

Inclusion/Exclusion

2. Revision of **inclusion criteria** such that filling out 50% of baseline daily questionnaires and a mean daily pain of 4 or above on the baseline daily questionnaire are no longer required for entry into study (4.1 and 4.2).
3. Due to change #2, it is no longer necessary to perform 2-step consent, which is replaced with **a single step consent** at the screening visit (4.3 and 6.2).
4. Changed **exclusion criteria** from ASA classification IV and above to ASA classification III and above to minimize risk to participants (4.2).
5. Replaced Beck's depression index with **MINI** (in the discretionary exclusion list) to better identify and assess psychiatric conditions that may interfere with the proper receipt of the study interventions. (4.2, 6.2)

Participant Recruitment

6. Added an **online screening form** so we can direct those who learned about our study online to visit our study immediately and know relatively soon if he/she is eligible (4.3.1).
7. Added r**escreen window of 4 weeks** to screening eligibility section (4.3.1).

Study Procedures

8. Revision of the **schedule of evaluations** to include personnel responsible to fill out each questionnaire/CRF (6.1), and revision of **study procedures** (6.2) to reflect

changes due to the transition **from 2-step to 1-step consent**, and to reflect the most up to date changes in each study visit.

9. Revised language when referencing **actigraphy** measures (6.1) by emphasizing the word “optional.” This test may be excluded from the protocol.

Randomization

10. Revised **randomization plan** from simple randomization to randomization stratified to each acupuncturist/site with a block size of 4, to account for the increased number of acupuncturists in the study to cover the greater Bay Area (6.2.2).
11. Added more **details on procedure of randomization** (6.2.2) to a) streamline the process; b) ensure blinding of the research team (the PI and research coordinator [RC]).

QA plan (deviations and AE’s).

Established standard procedure to QA the study: the PI and RC will perform **monthly QA checks** on: informed consents, participant surveys, research team CRF’s, acupuncturist’s CRF’s and treatment audiotapes. For each participant, protocol deviations and all forms of AE’s will be logged accordingly at a monthly basis on Excel spread sheet (7.4, and 10.3.5).

Version Number: 4.0

Version Date: 8/29/2016

Summary of Revisions Made:

In this revision, we widened both the inclusion criteria and the rescreening window in an effort to boost recruitment and maximize participant retention with minimal impact to the study quality.

Inclusion criteria (page 15): 1) decreased pain level from 5 or above to 4 or above out an 11-point (0-10) numerical rating scale (section 4.1); 2) decreased acupuncture-free period from the last decade to the last 5 years (section 4.2).

Participant rescreening (page 16 & 17): Currently many of our acupuncturists are at full capacity due to active engagement with the P01. Participants in this K23 study may need to wait for 6-10 weeks between the time of their screening visit and the first acupuncture treatment. It is thus of interest to expand the rescreening window to allow this waiting period. We increased the rescreening window from 1 to 3 months with following policy to facilitate the waiting period while being careful to ensure that the participant remains eligible (section 4.3.1).

a. Participant returns for pre-treatment (ie baseline) visit within 1 month after the screening visit: no additional screening necessary.
b. Participant returns between 1 and 3 months after the screening visit: rechecking mean pain score over 30 days, and medication list.
c. Participant returns between 3-6 months after the screening visit: rechecking mean pain score, medication list, PH-9, perform inclusion/exclusion CRF and screening physical exam.
d. Participant returns > 6 months after the screening visit, repeat the entire screening visit (all of the tests in step C, plus questionnaires, QST and HRV tests).

Version Number: 4.1

Version Date: 9/20/2017

Summary of Revisions Made:

This revision was made to correct minor errors identified by the WESTAT mid-trial visit from August 2017. The majority of the changes take place in section 6.1 (page 33) to reflect minor errors on the schedule of surveys/CRF's outlined in the table.

Additionally, for the inclusion criteria, both our communications with NCCIH and our IRB listed a minimum of 3 years since the most recent acupuncture treatment, but we mistakenly wrote down 5 years in version 4.0. This error was corrected in section 4.2 of this revision.

For participants who missed filling out more than 50% of the daily surveys, we will mark this as a protocol deviation (section 6.2.2) per suggestion by WESTAT.

Finally, one of the acupuncturists (AF) passed away this year and our administrative assistant (CD) is replaced by a new one (KH).

Version Number: 4.2

Version Date: 10/3/2017

Summary of Revisions Made:

This revision is a follow up to address TWO concerns raised by OCRA regarding two issues from version 4.1.

First, we would like to clarify on a mistaken assumption we've made regarding **our primary outcome, the PROMIS pain intensity instrument (PPII)**, which is collected at pre-treatment and post-treatment visits. We assumed it contained an **11-point numerical rating scale (NRS)** measure of mean pain intensity. However, in fact, it consists of 3 questions, assessing the mean, most and least pain intensity over the last 7 days, ALL on a **5-point Likert Scale** (no pain=1, mild pain=2, moderate pain=3, severe pain=4, very severe pain=5). The PPII is scored by adding the patient's response to all 3 questions (ranging from 3-15) and convert the raw score to a T-score (0-100) where 50 represents population mean and 10 represents standard deviation.

Because of the above change in the exact measure of our primary outcome (from 11-point NRS to 100 point T-score), we need to make the **following adjustment to the definition of a responder**. Previously, based on established literature (Farrar, Young et al, Pain, 2001), we defined a responder as those with ≥ 30% or ≥2 points of pain reduction (pre-treatment minus post-treatment) using the NRS. With the PPII, there isn't a universally agreed upon minimally significant difference (MID). However, Yost and Eton estimated the MID's of several PROMIS instruments in cancer patients (Yost, Eton et al, J Clin Epidemiol, 2011). These included MID of T-scores between 4.2 and 4.5 for pain measures. Thissen and Liu estimated the MID of

pediatric PROMIS instruments for depression, pain, fatigue and mobility, and found that the MID for these tend to cluster around a T-score of 2 for pediatric patients, and around 3 (2.4 to 3.5) for adolescents and parents (Thissen, Liu et al, Qual Life Res, 2016). Taken together, with limited available data, we feel that a T-score of 5 is a reasonable choice for MID for the PPII. Alternatively, a 1-point deduction on the 5-point Likert scale (1-5) for the MEAN pain intensity subscale (singe question) will be used to replace the 2-point reduction on the 11-point NRS (0-10). **In summary, with the PPII, we define the primary outcome as the change in T-score of the full 3-item instrument. A responder is defined as a completer who showed ≥ 5-point reduction in the T-score of the full instrument, OR, ≥ 1-point reduction in the 5-point mean pain intensity sub-scale.** For good measure, from here on, we will be adding the 11-point NRS to the PROMIS packet at both the pre- and post- treatment visits for ALL future participants. The above clarification/changes of primary outcome, responder definition and additional of the NRS scale will be reflected throughout this protocol, including the Precis section, and sections 1.2, 3, 6.1, and 9.5.1.

Last but not least, **we confirmed that our power calculation and statistical methods remain the same despite the slight adjustment in the measure of the primary outcome (pain).** Specifically, the power calculation does not change, because it is estimated using TS as our primary predictor for a moderate change in pain, measured either in NRS or the T-score from the PPII. Furthermore, there also will not be changes in our statistical methods, as the outcome measure remains to be percentage changes from pre-treatment, measured at post-treatment, regardless of the actual measure (0-10 in NRS or 0-100 in T-score).

Second, per OCRA's suggestion, we removed all languages related to Actigraphy. If units become available later in our study, we will add back Actigraphy with detailed plans of use.

Version Number: 4.3

Version Date: 10/19/2018

Summary of Revisions Made:

This revision was made to correct issues identified by the WESTAT interim monitoring visit from August 2018. Most of these changes involve following up on previously missed details and/or clarification of research procedures. These protocol revision will not result in any major changes in the conduct of this study.

1. Eliminate languages involving the MINI (pg 18 and 37). Due to a clerical oversight, we included MINI in some of our language, yet, we have NEVER used the MINI as our psychiatric screener. MINI had been only considered during our earlier planning phase and we had rejected it due to its cost and the time needed for its administration. Previous studies have used PH-9 successfully in the screening of suicidality and major depression. We have been using PH-9 since the start of our study.

2. Clarification on long-term follow up (pg 42, under “online follow-up.”) We have clarified on the exact timing of the 3, 6, and 12 month follow-up surveys, measured from the day of the last study treatment, and the windows for the follow up surveys, per suggestions from WESTAT.

3. Clarification on weekly surveys (pg 36 and 38). We have clarified that all weekly surveys during the study period have the same exact content. They are administered after the completion of the baseline (pre-treatment) visit on a weekly basis until the completion of the post-treatment visit. Greater than 50% missing of weekly surveys in an individual will be considered a protocol deviation.

4. Clarification on assessment of radicular pain symptoms on pg 18, section 4.2. One of the exclusion criteria in our study is the presence of radicular pain. Per discussion with WESTAT, we have the clarified the research procedures to rule out radicular pain.

5. Clarification on physical exam (top of pg 42). We have never performed physical exam at the post-treatment visit. Our protocol was accurate in the overall assessment table on page 36 but there was an error on the list of tasks for the post-treatment visit on p42. This error is now corrected.

6. Clarification – QI of acupuncture treatment audios (pg 32 and 33). Per request from WESTAT, we clarified that we will listen to 1 to 3 audio records from 20 randomly selected participants to ensure adherence to protocol.

7. Personnel update – see page 11 for changes in RC and in acupuncturist roster.

TABLE OF CONTENTS

Page

Clinical Intervention Study Protocol Template **1**

PREFACE **2**

FULL PROTOCOL TITLE **3**

Tool Revision History 4

TABLE OF CONTENTS **5**

STUDY TEAM ROSTER 8

PARTICIPATING STUDY SITES 8

PRÉCIS 8

1. STUDY OBJECTIVES **8**

1.1 Primary Objective 8

1.2 Secondary Objectives 9

2. BACKGROUND AND RATIONALE **9**

2.1 Background on Condition, Disease, or Other Primary Study Focus 9

2.2 Study Rationale 9

3. STUDY DESIGN **9**

4. SELECTION AND ENROLLMENT OF PARTICIPANTS **10**

4.1 Inclusion Criteria 10

4.2 Exclusion Criteria 10

4.3 Study Enrollment Procedures 11

5. STUDY INTERVENTIONS **11**

5.1 Interventions, Administration, and Duration 11

5.2 Handling of Study Interventions 12

5.3 Concomitant Interventions 12

5.3.1 Allowed Interventions 12

5.3.2 Required Interventions 12

5.3.3 Prohibited Interventions 12

5.4 Adherence Assessment 12

6. STUDY PROCEDURES **13**
6.1 Schedule of Evaluations 14
6.2 Description of Evaluations 15
6.2.1 Screening Evaluation 15
6.2.2 Enrollment, Baseline, and/or Randomization 15
6.2.3 Blinding 16
6.2.4 Followup Visits 16
6.2.5 Completion/Final Evaluation 17
7. SAFETY ASSESSMENTS **17**
7.1 Specification of Safety Parameters 17
7.2 Methods and Timing for Assessing, Recording, and Analyzing Safety Parameters 17
7.3 Adverse Events and Serious Adverse Events 17
7.4 Reporting Procedures 18
7.5 Followup for Adverse Events 18
7.6 Safety Monitoring 18
8. INTERVENTION DISCONTINUATION **18**
9. STATISTICAL CONSIDERATIONS **19**
9.1 General Design Issues 19
9.2 Sample Size and Randomization 19
Treatment Assignment Procedures 19
9.3 Definition of Populations 19
9.4 Interim Analyses and Stopping Rules 20
9.5 Outcomes 20
9.5.1 Primary Outcome 20
9.5.2 Secondary Outcomes 20
9.6 Data Analyses 20
10. DATA COLLECTION AND QUALITY ASSURANCE **21**
10.1 Data Collection Forms 21
10.2 Data Management 21
10.3 Quality Assurance 21
10.3.1 Training 21
10.3.2 Quality Control Committee 21
10.3.3 Metrics 21
10.3.4 Protocol Deviations 21
10.3.5 Monitoring 21

11. PARTICIPANT RIGHTS AND CONFIDENTIALITY 21
11.1 Institutional Review Board (IRB) Review.. 21
11.2 Informed Consent Forms 22
11.3 Participant Confidentiality 22
11.4 Study Discontinuation.. 22
12. COMMITTEES 22
13. PUBLICATION OF RESEARCH FINDINGS... 22
14. REFERENCES... 23
15. SUPPLEMENTS/APPENDICES 23
I. Procedures Schedule
II. Informed Consent Form Template
III. Other (add as many appendices as necessary)

STUDY TEAM

Name	Organization	Role on Project	Phone	Email Address	Office Address
Kong, Jiang-Ti	Stanford University School of Medicine	Principle Investigator	(650) 497-0493	jtkong@stanford.edu	1070 Arastradero Road, Suite 200, Palo Alto, CA 94304
Puetz, Chelcie	Stanford University School of Medicine	Research Coordinator	(650)724-8735	cpuetz@stanford.edu	1070 Arastradero Road, Suite 200, Palo Alto, CA 94304

Other Key Personnel

Name	Organization	Role on Project			
Langlois, Camille	Stanford Pain Division	Chief Acupuncturist	(510)708-8336	camille.langlois@gmail.com	3rd Floor, 1996 Union St, San Francisco, CA 94123
On, Showing	On Wellness Clinic	Acupuncturist	(415)508-5908	showing@onwellnessclinic.com	211 Sutter St, #502, SF, CA 94108

Eunyong Lee	Evergreen Acupuncture Clinic	Acupuncturist	(650) 922-2808	acupuncturemom@yahoo.com	77 Birtch St,Suite B, Redwood City, CA 94062
Reyna Ambrose	Fremont Acupuncture	Acupuncturist	(510) 789-0197	Reyna.ambrose.lac@gmail.com	39813 Paseo Padre Pkwy, Fremont, CA 94538
Kristen Honesto	Stanford Pain Division	Admin Randomizer	(650)723-1235	khonesto@stanford.edu	1070 Arastradero Rd, St 200, Palo Alto, CA 94304
Maria Preciado	Stanford Pain Clinic	Medical Assistant	(650)723-6238	mpreciado@stanfordhealthcare.org	450 Broadway Street, 1st Flr, Pavillion A, Redwood City CA 94063

PARTICIPATING STUDY SITES

Stanford Systems Neuroscience and Pain Laboratory (SNAPL)
1070 Arastradero Rd, Suite 200
Palo Alto, CA 94304
(650) 724-0525.

Community Acupuncture clinics in the Peninsula/San Francisco Bay Area.

PRÉCIS

Study Title

Predicting Analgesic Response to Acupuncture – A Pragmatic Approach.

A randomized, placebo-controlled, subject and assessor blinded, 100-subject clinical trial of electro-acupuncture in the treatment of chronic low back pain

Objectives

Aim 1. Explore the predictive relationship and the association between ascending sensitization measures and clinical response to acupuncture.

Aim 2. Explore the predictive relationship and the association between descending pain modulatory measures and clinical response to acupuncture.

Aim 3. Exploratory model building for predicting pain reduction by acupuncture.

Design and Outcomes

The current design is a randomized control trial in which 100 participants with chronic low back pain will be randomized with equal probability to verum (electro) acupuncture (VA) or sham/placebo (‘electro’) acupuncture (PA). Participants and researchers will be masked to treatment assignment; treatment providers will not (and cannot) be masked to treatment assignment.

Primary Outcome: Change in Pain Intensity score (T-score) measured by the PROMIS Pain Intensity Instrument administered at the pre-treatment and post-treatment visit.

Primary Predictors: temporal summation, conditioned pain modulation, pressure pain threshold, degree of widespread pain, pain catastrophizing, expectations, coping and pain self-efficacy.

Secondary Predictors: history of trauma, PTSD symptoms, illness-perception questionnaire, treatment appraisal questionnaire, depression, anxiety, fear-avoidance beliefs, heart-rate variability, heat pain threshold and tolerance.

Interventions and Duration

One hundred adult male and female participants with chronic, non-radiating low back pain (CLBP) will be randomly assigned to receiving either verum (VA) or sham/placebo (PA) electroacupuncture treatment with equal probability. The treatments will be delivered by 7 senior acupuncturists at 7treatment centers in the San Francisco Bay Area, within 60 miles from Stanford Hospital and Clinics. Each treatment will be around 45 minutes long, delivered twice per week, for a total of 12 sessions.

Participants will be assessed at screening, pre-treatment baseline visits, post-treatment visits, and at 3, 6, and 12 month follow-ups. Additionally, during the active treatment period, participants will be assessed daily for pain, sleep and function, twice per week at treatment, and weekly for treatment side effects.

Sample Size and Population

This project will recruit men and women with axial chronic lower back pain (greater than 3 months) and no radicular symptoms. Participants must be within the age range of 21 to 65. A total of 100 participants will be enrolled in the project.

1. STUDY OBJECTIVES

1.1 Primary Objective

Aim 1. Explore the predictive relationship and the association between ***ascending sensitization*** *measures and clinical response to acupuncture.*

Hypothesis 1: augmented temporal summation (TS), and more wide-spread pain (WSP) (quantified by number of painful areas marked by the participant on a standardized body map) will be associated with greater pain reduction in the verum arm than in the placebo arm; decreased pressure pain threshold (PPTr) will be associated with greater pain reduction in the placebo arm than the verum arm.

Aim 2. Explore the predictive relationship and the association between ***descending modulatory*** *measures and clinical response to acupuncture.*

Hypothesis 2: decreased conditioned pain modulation (CPM), and more favorable psychological state, measured by less pain catastrophizing, better self-efficacy in coping and in pain management, will be associated with greater pain reduction in the verum than the placebo arm; expectation of positive treatment outcome will be associated with pain reduction in both the verum and the placebo arm.

Aim 3. ***Exploratory model building*** *to predict pain reduction by electroacupuncture.*

Hypothesis 3. A mathematical model relating key baseline characteristics to percent pain reduction will be developed using advanced techniques, and this model will be tested using cross-validation within the proposed pilot.

1.2 Secondary Objectives

1. Alternative outcome measures: pain intensity measured by an 11-point NRS; physical functioning, measured by the PROMIS physical functioning scale will be assessed before and after the intervention, and the immediate percent change in physical functioning measured by the Roland Morris Disability Questionnaire, will be used as a secondary outcome. Furthermore, long-term relief, measured in both pain and physical functioning will be used as additional outcome measures in the analyses performed in Aims 1, 2 and 3.

2. Exploring objective markers: heart rate variability is sometimes associated with wellness. For example, increased variability in the low frequency spectrum is considered a measure of robust parasympathetic system and good health.[1] A few small studies has shown effects of acupuncture on improving heart-rate variability.[2-4] HRV will be captured prior to and immediately after the interventions.

3. Characterizing response to acupuncture at high resolution: the daily symptom capture tool included in this project will allow characterization of clinical response (pain and symptom improvement) at a higher granular level than previously possible, to the best of our knowledge.

2. BACKGROUND AND RATIONALE

2.1 Background on Condition, Disease, or Other Primary Study Focus

100 million Americans suffer from chronic pain.[6] Conventional treatments are often inadequate.[6] Acupuncture reduces pain better than conventional care (ie medications, physician and physiotherapy visits) for several pain conditions.[7] However, not all patients respond to acupuncture,[8] and it often takes multiple sessions to determine if

acupuncture is effective. Thus, there is a great need in capturing and understanding factors that may predict clinical response to acupuncture analgesia.

The characteristics that differentiate responders from non-responders to acupuncture analgesia remain elusive. Although acupuncture studies have evaluated patient demographics, comorbidities, and expectations, most of these turned out to be insignificant predictors,[9-14] except for expectation which yielded mixed results.[10,15,16]

Researchers have rarely explored predictive factors that are unique to chronic pain. Pain is influenced by the integration of both ascending nociceptive signals and descending cognitive-emotional modulation at key relay centers.[17,18] In chronic pain, the ascending transmission of pain is often augmented, which may be considered as ascending sensitization.[19] In contrast, descending inhibition of pain is often reduced.[20] Standardized quantitative sensory testing (QST) can measure changes in both the ascending and descending pain pathways,[21] whereas psychological processes mainly contribute to the descending regulation of pain.[22] Although both QST and psychological assessments are common diagnostics in pain research, they have not been systematically examined in the context of predicting acupuncture analgesia.

Taking advantage of the latest studies in personalizing pain medicine where select QST and relevant psychological measures were found to be potential predictors of long term outcome in chronic low back pain (see section 2.2 below), we will explore the associations between these factors, and the outcomes to electroacupuncture in treating back pain patients.

2.2 Study Rationale

1. Pressure Pain Threshold Predicted Pain Reduction by Sham Acupuncture in Patients with Fibromyalgia (Harte et al 2013 Medical acupuncture).[23]

Rationale: to determine if experimental pressure pain assessment and chemical neuroimaging can identify differential responsiveness to sham vs traditional acupuncture

Study: Fifty patients with fibromyalgia were randomized to either 9 traditional (TA) or sham (SA) acupuncture treatments over 4 weeks. Both participants and assessors were blinded.

Results: Patients with low pain sensitivity (LPS), but not with high pain sensitivity (HPS), had a significantly reduced clinical pain response to SA (change in mean [standard deviation (SD)]: HPS - 8.65 [7.91]; LPS - 2.14 [6.68]; $p = 0.03$). This relationship was not the case for TA (HPS - 6.90 [4.51]; LPS - 6.41 [9.25]; $p = 0.88$). SA-treated patients who were more sensitive also had greater baseline levels of insular Glx than patients who were less sensitive (Glx mean [SD]: HPS 11.3 [1.18]; LPS 10.2 [0.54]; $p = 0.04$).

Relevance: this study demonstrates the potential for pressure pain sensitivity, a measure that will be captured in our study, in differentially predicting response to sham vs verum acupuncture in patients with chronic pain.

2. Conditioned Pain Modulation Predicted Response to Duloxetine in Patients with Diabetes Peripheral Neuropathy (Yarnitsky 2012 Pain).[24]

Rationale: to determine if pain modulation patterns as assessed by temporal summation (TS) and conditioned pain modulation (CPM) may predict clinical response to Duloxetine, a serotonin-norepinephrine reuptake inhibitor

Study: Thirty patients with painful diabetic neuropathy received 1 week of placebo, 1 week of 30 mg/d duloxetine, and 4 weeks of 60 mg/d duloxetine. Pain modulation was assessed psychophysically, both before and at the end of treatment. Patient assessment of drug efficacy, assessed weekly, was the study's primary outcome. Results: Baseline CPM was found to be correlated with duloxetine efficacy ($r=0.628$, $P<.001$, efficient CPM is marked negative), such that less efficient CPM predicted efficacious use of duloxetine. Regression analysis ($R(2)=0.673$; $P=.012$) showed that drug efficacy was predicted only by CPM ($P=.001$) and not by pretreatment pain levels, neuropathy severity, depression level, or patient assessment of improvement by placebo. Furthermore, beyond its predictive value, the treatment-induced improvement in CPM was correlated with drug efficacy ($r=-0.411$, $P=.033$). However, this improvement occurred only in patients with less efficient CPM (16.8 ± 16.0 to -1.1 ± 15.5, $P<.050$). No predictive role was found for TS.

Relevance: the coupling of CPM and duloxetine efficacy highlights the importance of pain pathophysiology in clinical decision making. CPM and TS will also be examined in the current study. We hypothesize that because electroacupuncture will influence both descending pain modulation (measured by CPM) and ascending pain facilitation (measured by TS), both measures will be predictive of pain reduction by acupuncture.

3. Degree of widespread pain was associated with clinical outcome to acupuncture: An Observational Pilot.

Rationale: to determine if widespread pain at baseline may predict pain reduction by acupuncture

Study: In collaboration with the NIH Pain Consortium, we recently began an open source platform to capture real-world, patient-reported outcomes (PROs) at the Stanford Pain Management Center. This platform includes an interactive BodyMap for patient to indicate the locations of their pain. This BodyMap can be used as a surrogate measure of widespread pain. The more areas marked off by a patient as painful, the more widely spread is his/her pain. We define an acupuncture course as consecutive sessions no more than two weeks apart. We used Fisher’s exact test to evaluate whether increase areas in BodyMap marked by the patient predicted clinical response to acupuncture (≥ 30% pain reduction).

Results: Our system captured 34 patients with pain rating and BodyMap data within 30 days prior and 30 days following the acupuncture course. About 40% of the 34 patients responded by reporting at least 30% reduction of pre-acupuncture pain. Importantly, we found a trend that widespread pain defined by ≥ 6 BodyMap areas predicted pain reduction by acupuncture with an odds ratio of 3.4 (p-value 0.33 by Fisher's exact test).

Relevance: the preliminary results in this pilot suggests the possibility that widespread pain may potentially predict responsiveness to real-world acupuncture

treatments. Recognizing the low sample size at this stage, we will be pursing similar type of analysis in a larger sample size both in a real-world setting at our clinic to improve patient care, and, in the current clinical trial to be initiated soon.

4. Multiple psychological factors are associated with long term outcome in back pain patients receiving acupuncture (Bishop 2015, Clin J Pain).[25]

Rationale: To identify psychological covariates of longitudinal changes in back-related disability in patients undergoing acupuncture.

Study: A longitudinal postal questionnaire study was conducted with data collection at baseline (pretreatment), 2 weeks, 3, and 6 months later. A total of 485 patients were recruited from 83 acupuncturists before commencing acupuncture for back pain. Questionnaires measured variables from 4 theories (fear-avoidance model, common-sense model, expectancy theory, social-cognitive theory), clinical and sociodemographic characteristics, and disability. Longitudinal multilevel models were constructed with disability over time as the outcome.

Results: Within individuals, reductions in disability (compared with the person's individual mean) were associated with reductions in: fear-avoidance beliefs about physical activity ($\beta=0.11$, $P<0.01$) and work ($\beta=0.03$, $P<0.05$), catastrophizing ($\beta=0.28$, $P<0.05$), consequences ($\beta=0.28$, $P<0.01$), concerns ($\beta=0.17$, $P<0.05$), emotions ($\beta=0.16$, $P<0.05$), and pain identity ($\beta=0.43$, $P<0.01$). Within-person reductions in disability were associated with increases in: personal control ($\beta=-0.17$, $P<0.01$), comprehension ($\beta=-0.11$, $P<0.05$) and self-efficacy for coping ($\beta=-0.04$, $P<0.01$). Between individuals, people who were less disabled had weaker fear-avoidance beliefs about physical activity ($\beta=0.12$, $P<0.01$), had more self-efficacy for coping ($\beta=-0.07$, $P<0.01$), perceived less severe consequences of back pain ($\beta=0.87$, $P<0.01$), had more positive outcome expectancies ($\beta=-0.30$, $P<0.05$), and appraised acupuncture appointments as less convenient ($\beta=0.92$, $P<0.05$).

Relevance: this study demonstrated the importance of psychological factors and beliefs in influencing the long term outcomes in patients with chronic back pain who also received acupuncture. Note this is an uncontrolled association study. Our current study will re-examine the key predictors uncovered in this paper in a controlled, blinded trial.

3. STUDY DESIGN

This is a randomized placebo-controlled trial in which 100 consented adults with chronic low back pain (CLBP) will be randomized to 6 weeks of twice a week sessions with verum acupuncture (VA) or sham/placebo-acupuncture (PA). Participants and researchers will be blind to treatment assignment. Treatment providers will not (and cannot) be masked to treatment assignment.

Timeline: Eligible participants will be invited for a baseline visit. Since the focus of this study is prediction, extensive collection of baseline characteristics, including physical (vital signs and heart rate variability, physical exam), psychological (questionnaires), and QST measures will be collected around and at the time of the baseline visit. Approximately 1 week after the baseline visit, the participant will enter

a 6-week treatment phase, during which they will be randomized to VA or PA. (See Section 4.3.2. for details of randomization). The Post-Treatment Visit will take place 1 to 2 weeks after the last treatment visit. Participants will then be followed up and assessed at 3, 6 and 12 months after treatment ends. Thus, participants will be in the project for a total of approximately 12 months.

Location: The treatments will be provided at 7 sites: our laboratory (SNAPL) where our main acupuncturist (Camille Langlois Loveman) will be providing services to patients located on and near the Peninsula; as well as 6 other acupuncture clinics located in the greater San Francisco Bay Area (SF, South and East Bay). The baseline and post-treatment visit will take place at our laboratory, SNAPL (Systems Neuroscience and Pain Laboratory) at the Stanford Division of Pain.

Interventions: The interventions, verum (electro) acupuncture (VA) and sham/placebo (‘electro’) acupuncture (PA), are described in detailed in Section 5 (Project Interventions). Interventions will be provided at no charge to participants.

Primary outcomes: a) Change in Pain Intensity measured by the PROMIS Pain Intensity Instrument administered at the pre-treatment and post-treatment visit. . b) a responder is defined as one who experiences ≥ 10% reduction in the T-score (1 standard deviation) of the full instrument, or ≥ 1 point decrease in the 5-point Likert Scale of the mean pain intensity question in the PROMIS Pain Intensity Instrument.

Secondary outcomes: Pain ratings from an 11-point numerical rating scale (NRS), functional capacity measured by the Roland Morris Disability Questionnaire and the physical function instrument from NIH PROMIS.

4. SELECTION AND ENROLLMENT OF PARTICIPANTS

A total of 100 participants, age 21-65, with chronic axial low back pain for at least 6 months, will be enrolled.

4.1 Inclusion Criteria

Participants must meet all of the following inclusion criteria in order to be enrolled in the project.

- Males and females, ages 21-65
- English fluency
- Have had chronic low back pain for at least 6 months:

Definition of Chronic Low Back Pain (cLBP)—criteria from the NIH Task Force for cLBP

  - Chronicity: cLBP “defined as a back pain problem that has persisted at least 3 months and has resulted in pain on at least half the days in the past 6 months.”
  - Location: "between the lower posterior margin of the rib cage and the horizontal gluteal fold.”

- Average pain over the last month ≥ 4/10 for initial eligibility

4.2 Exclusion Criteria

All candidates meeting any of the exclusion criteria at screening will be excluded from the project.

- Radicular low back pain: defined as sharp (or burning) pain, with a defined territory, radiating down the limb, beyond the knee. Diagnosing radicular pain in the absence of an electromyogram (EMG) or MRI is a challenging problem, because referred pain from axial structures (such as the ligmentum flavum and lumbar facets) may involve the legs or even the feet, thus mimicking radicular pain. In order to accurately identify radicular pain while minimizing false positives, we use the following criteria: 1-the participant must report frequent symptoms of spontaneous radicular pain as defined above (at least once a week) accounting for at least 30% of his/her total pain complaint; AND, 2- the participant must demonstrate a positive response to a dorsiflexion test during the screening physical exam. A dorsiflexion test is a clinical exam performed in the supine position. While keeping one leg flat, the experimenter raises the other leg of the participant (with a straight knee) to near the flexibility limit, at which point the experimenter dorsiflexes the foot of the participant's raised leg. This test is deemed positive if the participant reports true radicular pain (sharp, well-demarcated, shooting pain that travels beyond the knee) upon dorsiflexion. Given the challenging nature of this exam, we have provided extensive training of our RC's via clinical papers, videos and multiple practice sessions. In addition, we allow the RC's to consult the PI (MD) if ambiguities arise from the history and the physical exam. Ultimately, for ambiguous cases, the PI will make a final decision on the presence of radicular pain/symptoms based on clinical judgment.
- Pending litigation or Worker's compensation related to the low back pain.
- Currently pregnant or planning to become pregnant
- American Society of Anesthesiologist (ASA) class III or above physical status. ASA class III is defined as "a patient with severe systemic disease." Examples would include poorly controlled diabetes, hypertension, COPD or morbid obesity (BMI ≥ 40).
- Mental health conditions or treatment for mental health problems that would interfere with study procedures, at the discretion of the study team. For example, psychosis, untreated major depression, ongoing substance abuse, suicidal ideation. These will be assessed by the PH-9.
- Medications: opioids ≥60mg morphine equivalent units/day, benzodiazapenes, corticosteroids.
- Prohibited interventions: during the study period, the following are not permitted
    - Back surgeries
    - Injections with local anesthetics or steroids to the back
    - New chiropractic maneuvers
    - New physical therapy programs
    - New medications for back pain
- Bleeding disorders at the discretion of the study team.
- Previous acupuncture treatment in the past 3 years.
- Medical conditions that would interfere with study procedures (eg. Heart disease or pacemaker, active infection), per discretion of the team

4.3 Study Enrollment Procedures

4.3.1 Recruitment

Stanford Pain Clinic: About 50% patients at the Stanford Pain Center who are referred to acupuncture were denied by their insurance to receive this service. The clinic admin (M. Preciado) will provide these patients information on the K23 study (she will have IRB approved study brochures at the clinic to provide to patients upon request), and keep a list of the patient who expressed interest and consented to be contacted by the research team. The research team will access this list regularly (once every 1-4 weeks) and reach out to these patients.

Stanford CAM Center for Back Pain (database sharing with NCCIH P01 trial): The Stanford CAM back pain center has a large database of patients with back pain. Because the P01 trial is a mechanistic study and has stringent inclusion criteria, the majority of the individuals from the P01 database are ineligible for P01 because of comorbid medical conditions, medication use, or MRI contra-indications. Our research team has access to a log on the eligibility details of patients on this data base and will be able to rapidly screen these patients for the K23 study, which has much less stringent inclusion criteria than the P01 study.

Clinicaltrials.gov posting: The research team will register the K23 study on both the Stanford Clinical Trial site and clinicaltrials.gov. Interested patients may contact the team at the contact information provided.

Craigslist ads: Monthly Craigslist ads will be placed to advertise for the project. These ads will include basic details about the project and contact information for the project Recruiter.

Flyers, recruitment cards, and brochures in the community: We will be distributing project flyers, recruitment cards, and brochures throughout the community at locations such as cafes, restaurants, yoga studios, gyms, YMCAs, libraries, et cetera.

Community clinics: The PI (JT Kong) will send emails to colleagues in the community pain clinics as well in the primarily care colleagues in the Peninsula and SF Bay area to solicit appropriate patients for the study.

Online Screening Form (OSF): Interested participants from all of the above sources will be directed to a secure, online screening form that gathers basic contact info and back pain info. The research team will manually review entries from this REDCAP form on a minimally weekly basis, and contact those who are eligible from this online form.

Phone Screening and Screening Logs

Participants who have passed the OSF will be screened over the phone to further determine eligibility by the research coordinator (RC). A record of all phone screens will be kept in the Screening Log including phone screen result, reason for ineligibility, and reason of lack of interest by the participant. All phone screens conducted will also be entered into the SNAPL Central Database, from which it will be possible to retrieve this data to assess why callers are ineligible.

Eligible and Interested Participants

Eligible and interested participants will be invited to our site for a Screening Visit. At this visit, if participants are eligible, they will be consented for the entire study. They will be explained the details of the study objectives, interventions as well as all necessary assessments.

After the initial screening visit, the participant will be asked to fill out brief daily questionnaires on their back pain symptoms for 1-2 weeks before they come in for a baseline visit which will take place immediately before initiation of treatments. Between the screening visit and the baseline visit, if participants indicate that they have developed an illness, have travel plans or may not complete the baseline and treatment visits as outlined, their participation will be put on hold and they will be invited to participate in the study again after the resolution of the issues.

Rescreening window: in addition to participant side issues, our acupuncturists sometimes experience full capacity from another study (P01, AT006651). As such, participants may need to wait for up to 6-10 weeks before slots open up. To accommodate such waiting time, we’ve expanded our rescreening window (defined as time between the screening and the pre-treatment visit), from 1 month to 3 months, with the following policy to ensure that the participant remains eligible after waiting:

1. Participant returns within 1 month after the initial screening visit: no additional testing needed at the pre-treatment visit.
2. Participant returns between 1 to 3 months after the screening visit: At the beginning of the pre-treatment visit, confirm eligibility by repeating question on mean-pain over the last 30 days, and re-check medication list. If new or increased dose in pain meds, repeat inclusion/exclusion CRF (by the RC) and physical exam. If new or increase dose in psychiatric meds, repeat PH-9 questionnaire.
3. Participant returns between 3 to 6 months after the screening visit: At the beginning of the pre-treatment visit, confirm eligibility by repeating mean-pain over 30 days and PH-9 questionnaires (by participant), repeat medication and inclusion/exclusion CRF (by RC), repeat physical exam.
4. Participant returns more than 6 months after the screening visit: repeat the entire screening visit. Proceed to pre-treatment visit per protocol (ie 1-2 weeks later, and ONLY IF participant passes the repeat screening visit).

Participants not Interested or Ineligible

Participants who are not interested or ineligible will be asked if they want their information to be included in the SNAPL Central Database so that they may be contacted for future projects that are of interest to them.

Database Registration

The Research Staff will register all consented participants into the SNAPL Central Database with their unique project ID number and demographic information.

Recruitment Contingency Plan

We have a recruitment plan in place to ensure steady enrollment. This includes recurring advertisements on Craigslist, local newspapers, direct mailers, and a presence on the social media

(the research team will consider a Youtube video). The research team will be contacting community clinics, primary care physicians, free clinics, yoga studios, gymnasiums, and other such facilities to give talks on the Center for community outreach and to educate and build relationships with practitioners who can refer participants to us.

4.3.2 Randomization

When a new participant has passed the initial screening and baseline visit, he/she will be randomized to receiving either VA or PA with equal chance. An R program for randomization will be used. We will randomize patients within each of the 7 acupuncture sites with equal chance of receiving either VA or PA. Our lab manager or his associates (who are NOT immediately related to the proposed project) will run the randomization program and inform the treatment assignments to each provider via secure emails. Note that the participant will be able to pick the acupuncturist based on ease of access (commute time). Lastly, block randomization will be employed within each of the 7 acupuncturists to minimize chance imbalance in the number of verum vs sham assignments for each provider in case there is a large difference in the number of patients assigned to a provider (we observed this situation in the current P01 trial).

4.3.3 Consent

Consent Process: Prior to conducting any study procedures, designated and trained research staff will review the IRB-approved Consent and HIPPA forms with the participant in a private area, answer any questions, witness informed consent and the date obtained, and verify by signing as the research team member that obtained consent. Each participant will receive a signed and dated copy of their Consent Form and HIPAA documents.

The consenting process will take place at the screening visit. At the beginning of the screening visit, the participant will be consented for the research project. The consent process will include explanation of the details of the study, including interventions, visits, assessments as well as the timeline. Furthermore, participants will also learn about our HIPAA compliant information collection procedures and sign the HIPAA form. Research staff will answer all project-related questions and make sure that the participant fully understands all procedures, tests, and visits for the project**.** Participants will be given all the time necessary to comprehend the research project and procedures and all of their questions will be answered prior to them signing and dating the consent and HIPAA form. They will also be given the opportunity to receive a signed copy of their consent forms.

Storage of Consent Forms: Consent and HIPPA forms will be stored in a dated binder labeled "PHI and Consent Forms for Acupuncture Predictor Study". The binder(s) will be stored in locked cabinets in Dr. Kong's lab at 1070 Arastradero Road, Suite 200, Palo Alto, CA 94304. Only research staff will have access to study documents. Each consent form will be labeled in the bottom left corner with subject ID number.

5. STUDY INTERVENTIONS

5.1 Interventions, Administration, and Duration

5.1.1 General Concerns

Verum acupuncture (VA): to be administered by private licensed acupuncturists in their respective private offices. Each acupuncturist has over 3 years of practice experience. The treatment sessions will be around 45 minutes long out of which 20-25 minutes will involve active electrical stimulation of the needles. Within each session, in addition to body acupuncture, each participant will also receive standardized heat and auricular acupuncture therapy. The treatments will be delivered twice a week for 6 weeks, making a total of 12 treatments. We allow up to 3 missed sessions total (25%), two of which may be consecutive.

Sham/placebo acupuncture (PA): will also be administered by the same acupuncturists who deliver the verum acupuncture. PA treatments will follow the same exact time course as the VA treatments (around 45 min per session with 20-25 min under sham stimulation, 2 sessions per week for 6 weeks) and subject to the same rules for missing sessions. As with VA, participants assigned to the PA arm will also receive heat and auricular acupuncture therapy (both simulated, see detail in section 5.1.3) in each treatment session. Table 1 below summarizes the key parameters of each treatment. We designed these protocols to balance between increasing credibility of the PA and optimizing the therapeutic effect of the VA.

Table 1 Contrasting the Key Elements of the VA and PA Treatment Protocols

TREATMENT PARAMETER	VERUM (SUN) ARM	PLACEBO (MOON) ARM
Number of sessions	12	12
Frequency of sessions	2 / week	2 / week
Session Duration	45min	45min
Number of needles used	20	8
Flexibility of point selection	Yes. (Section 5.1.2.B) Based on anatomy (at baseline) and response (after 4 sessions);	Not flexible (Section 5.1.3.B)
% of points penetrated	100%	0% (all non-specific)
% of points hooked to wires	50% (all intact electrical wires)	100% (all broken wires for shamming)
Titration of the intensity of electrical stimulation	Real (see Section 5.1.2.C)	Simulated (see Section 5.1.3.C)

Heat Therapy	Standardized, to back, 30-40min	Standardized, away from back, 10-15min
Auricular acupuncture	4, fixed, pain specific points, with penetrating needles	2, fixed, pain NON-specific points, with non-penetrating tapes
Communication between acupuncturist and participant	Minimized and standardized	Minimized and standardized
Moxibustion	No	No
Music	No	No
Massage	No	No
Aromatherapy	No	No

5.1.2 Details of VA Administration

A. Rationale and Treatment Modules

The VA treatment protocol aims to treat low back pain with both local (anatomy driven) and distal (energetic, meridian driven) approaches. It has two principal modules (1 local, 1 distal) and two optional modules (also 1 local, 1 distal).

The principal modules consist of Module #1 and #2, as shown below in Figure 5 and Figure 6, respectively. Both are activated in ALL treatment sessions. Module #1 represents the local approach. It is a modified PENS (percutaneous electrical nerve stimulation), where key Bladder back Shu points in the affected dermatomes are needled and electrically stimulated (see "Ghoname EA, Craig WF, White PF, et al. *JAMA* 1999; 281(9): 818-23" for details of the original protocol).[26] Module #2, the distal approach, aims to tonify Shaoyin-Taiyang meridians by needling the following points: KI-3, KI-7, HR-3, SI-3, BL-40. Out of these, KI-3 (negative) and KI-7 (positive) are electrically stimulated as a pair. Two additional points, GV-3 and GV-20 will be needled to tonify Governor's Vessel meridian.

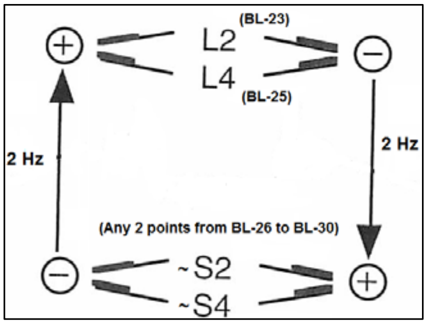

Figure 5 - Module 1: Modified PENS (obligatory)

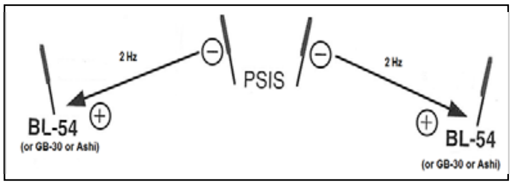

Figure 6 - Module 3: Periosteal (optional)

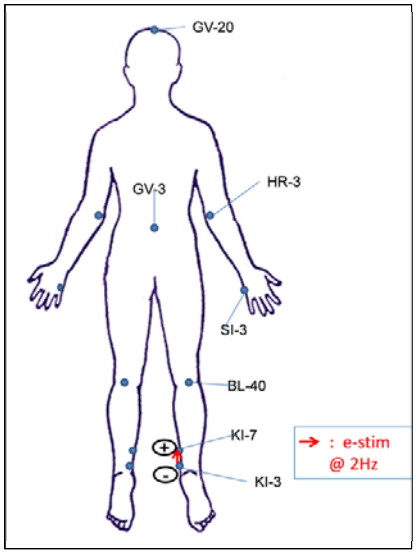

Figure 7 - Module 2: Principle Meridians (Obligatory)

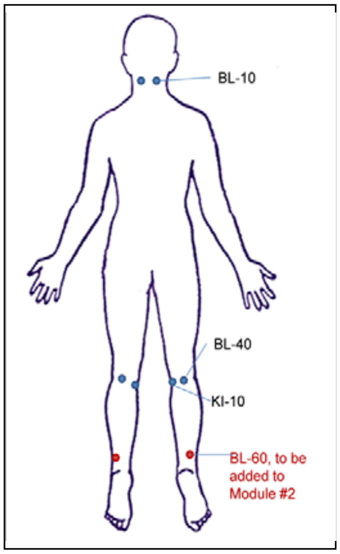

Figure 8 - Module 4: Distinct Meridian Activation (optional)

The optional modules are #3 (local) and #4 (distal). First, at baseline, if the back pain radiates to the buttocks and hips, we will activate module #3, the periosteal protocol for all session. It involves needling and paired stimulation of the posterior superior iliac spine (PSIS) and a deep muscular point in the affected gluteal region.[19] The posterior superior iliac spine is identified by palpating the posterior termination of the iliac crest (Figure 9). Second, if, after four treatments, the participant does not respond (see Section B below for criteria of clinical response), the kidney-bladder distinct meridian (module #4) will be activated for sessions 5-16. Distinct meridians are often needled to activate deep meridian energy in treating refractory conditions. The kidney-bladder distinct meridian consists of bilateral KI-10, BL-10, and BL-40 (which is activated in the principle treatment). Since BL-40 will be taken

out from Module #2 to complete Module #4, BL-60 will be added to complete Module #2. The activation of Module #4 does not involve electrical stimulation.

Last but not least, in addition to the above main building blocks, the VA protocol also includes 2 standardized elements in each treatment session: heat and auricular acupuncture. We made the decision to include standardize heat and auricular therapies to both mimic real-life practice of

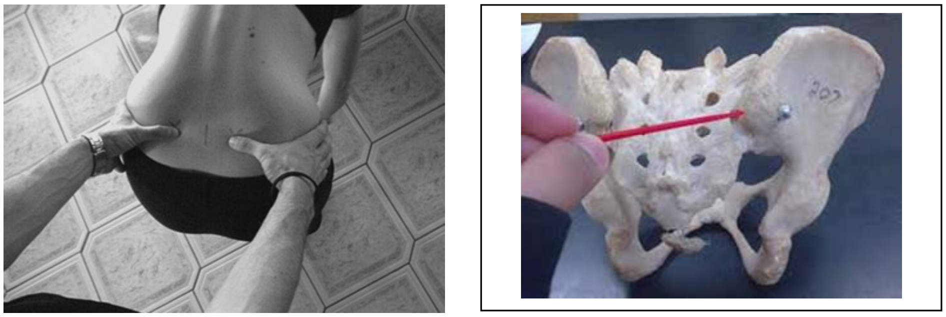

Figure 9 - Surface and bony landmarks of the posterior superior iliac spine (PSIS)

acupuncture and optimize the treatment effects.

B. Treatment flexibility and decision algorithms

Flexibility in VA

We will allow very little flexibility in the treatment for the proposed clinical trial because of three reasons. First, in contrast to many other clinical trials, our primary interest is in the mechanism of electroacupuncture (EA). As such, the more unified the treatment approach, the easier it will to identify a common mechanism underlying electroacupuncture. Second, Helm's approach to EA allows very little variation because: 1) the modified PENS uses a standard protocol; 2) the protocol assumes most CHRONIC low back pain is due to a combination of blockage of Qi in the bladder meridian and vacuity in the Kidney organ, further limiting the TCM choice points. Third, previous studies have shown, at least in the case of low back pain, the choice of points may not matter as long as local and distal points from the bladder and kidney meridians are stimulated.[20]

Decision algorithms

Although minimal flexibility is permitted, there are still TWO specific times during the treatment period that the acupuncturist will make a decision regarding WHICH TREATMENT MODULES to activate for subsequent sessions (Figure 10). Modules #1 and #2 will be activated in ALL treatment sessions. On session 1, depending on whether or not the pain radiates to buttocks and hips, Module #3 may be activated. On session #5, we will assess if the participant has responded clinically to the first four treatments. A response is defined as either ≥ 30% reduction of original pain rating or > 2 out of 10 points on the visual analog scale (VAS) of pain. If there is a response, the previous regimen will be continued. If not, we will activate Module #4 for the remainder of the treatment sessions (sessions 5 to 16).

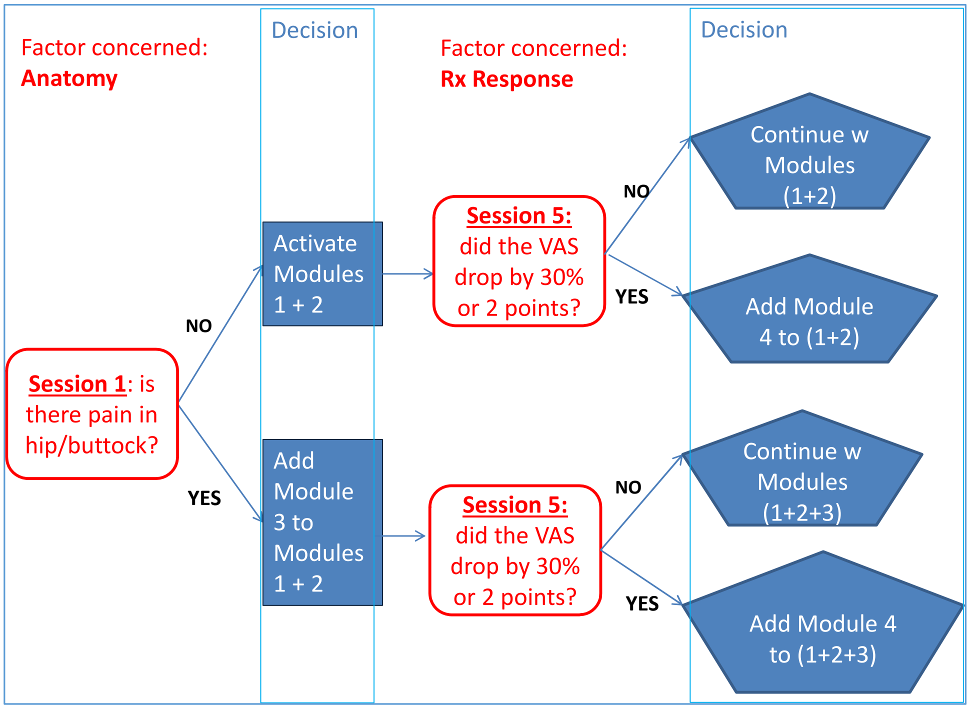

Figure 10. **Decision algorithms within the VA protocol (made by acupuncturist).** Module 1 = modified PENS (local); Module 2 = KD-BL meridian tonification (distal); Module 3 = periosteal (local); Module 4 = distinct meridian (distal). In addition to these main treatment modules, standardized heat and auricular therapy are applied at the beginning of all verum sessions.

Finally, we do not expect the treatment algorithm above to unblind the participants in the verum arm because: A) At the initial evaluation with the acupuncturist, all participants will be informed that they will receive treatments which may or may not vary from time to time depending on their specific conditions; B) BOTH VA and PA participants will be filling out STANDARDIZED questionnaires assessing their treatment response as part of the trial data collection; C) The additional module (module 4) is based on TCM principles, and does NOT involve electrical stimulation NOR additional points in the back. As such, unless the patients are well-versed in TCM, these 3 additional points should NOT draw extra attention from the participants leading to unblinding; D) On the other hand, the PA patients will be subject to the same rigorous evaluations, rituals, except they won't have the extra mystery points. Again, unless the patient is well versed in TCM theory and practice, the chance of unblinding the PA folks should be also minimal.

C. Stimulation parameters

Frequency

Ghoname et al showed previously that even though low frequency (2-4 Hz) for the PENS stimulation was sufficient to produce clinically significant pain relief,{Ghoname, 1999 #36} an alternating frequency of 15/30Hz produced even better results.{Helms, 1995 #37} On the other hand, stimulation of distal points (KI-3 and KI-7) has been traditionally carried out at lower frequencies (2Hz) to maximize tonification{Chen, 1992 #38} and to induce increase in CNS endorphin level.{Han, 2004 #39;Thomas, 1994 #40} Given this is a mechanistic protocol, the fewer variables we include the easier it would be to identify a mechanism. As such, we will use **2Hz for ALL stimulated pairs**.

Intensity

Several lines of evidence contributed to our decision to titrate the intensity of stimulation to "**visible muscular twitching at a level that the participant can comfortably tolerate**." First, the studies by Thomus[14] and Chu[15] identified muscular twitching as a predictive factor of response to treatment. Second, we ran a preliminary best practice survey on treating CLBP by EA in a select group of senior teaching acupuncturists. Out of the 27 respondents, all mentioned they would titrate to the participant's comfort level and about half described objective confirmation of muscular twitching. Third, the Helms method also calls for such combination of subject comfort and objective muscle twitching. If it is not possible to achieve muscular twitching, we titrate to the maximum stimulation intensity that the participant can comfortably tolerate for 25 minutes.

Stimulator and needle specifications

A direct current, square-wave electro-stimulator from Ito Co Ltd (Tokyo, Japan), model IC-1107+ will be used. It has three bipolar channels, with frequencies ranging from 1Hz to 100Hz, and delivers squared pulse waves with a width of 100 µsec. The maximum current output is 14mA +/- 20%.

For all points except for the deep muscular point in Module #3, all needles should be identical. Specifically, these will be Spring Ten sterile needles from DBC™. They are 30mm long and 0.2mm in diameter. Deqi sensation should be elicited for EVERY needle with or without electrical stimulation.

C. Additional Therapies

As indicated in Figure 10 above, each verum treatment is consisted of both individualized treatment modules (major) and standardized heat and auricular therapy (minor). In real life practice, acupuncturist often use simple adjuncts to augment their treatment effects. The Helm's protocol calls for the addition of both heat to low back and simple auricular needling which are applied at the beginning and maintained throughout each treatment session. In order to mimic real life practice and ensure maximum treatment effect, we opted to include these two modalities per the Helms protocol.

Heat: To ensure adequate heat is applied to the back for at least 30min, we will turn on the heat lamp as soon as participant is positioned in the prone position, before any needle placement. We will keep the heat about 30-40cm away from the skin to ensure close heating yet minimize chance of burning.

Auricular acupuncture: we will use 40G auricular needles to the follow points shown in Figure 11: Shenmen, Point Zero, Thalamus, and lumbar spine. We will place ear needles immediately after the heat lamp is on, before placing the first main needle, GV-20.

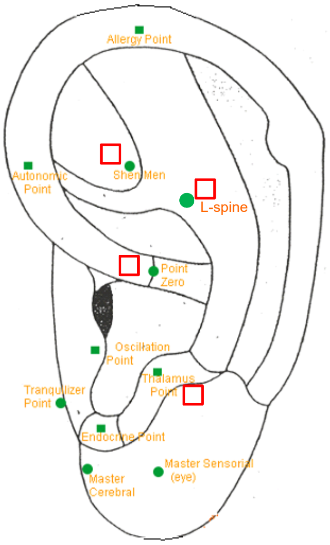

Figure 11 - Obligatory Auricular Points

D. Order of tasks for each VA session

After the patient is placed in the prone position, the heat and auricular modules (minor) are activated, the placement of the body needles should then start. The goal is to allow the PENS module (and the periosteal module, if included) to be stimulated for 20-25 minutes and the principle meridian (KI 3 to KI-7) to be stimulated for 15 minutes. Furthermore, GV-20 should be placed first (immediately after auricular needles are placed) to help with sedation.

Therefore, the order of actions should be:

Prone positioning, heat application, placing auricular needles,

GV-20, PENS, periosteal, start e-stim for both PENS and periosteal,

Shaoying-Taiyang circuit +/- distinct meridian circuit (if included), finally start e-stim for KI-3 to KI-7.

5.1.3 Details of PA Administration:

A. Rationale for design and point selection

In designing the sham treatment (PA), we balanced 2 objectives: a) to maximize credibility of PA; and b) to simultaneously minimize possible specific therapeutic effect from tactile stimulation of relevant areas on the body. To achieve the first objective, we used a generous number of needles (8 Streitberger needles), ensured the length and frequency of the treatment sessions in PA are equal to VA, and followed the same rituals in PA as those in VA, including titration of the sham electrical stimulation and the inclusion of heat and auricular therapy (both shammed in PA). To achieve the second objective, we used non-penetrating, Streitberger needles only, applied no electricity to the Streitberger needles by

intentionally breaking the wires to the electrical stimulator, and ensured the sham points are off-meridian and away from the focus of the patients' maximum pain.

As shown in Figure 12, the sham treatment consists of 8 sham points (bilateral S1 to S4) away from all meridians onto which the Streitberger apparatus will stand. To ensure the credibility of our treatment, we included both truncal (S2, S3) and extremity (S1, S4) sham points, all at least 2cm away from known meridians. Furthermore, S2 and S3 are away from patient's indicated area of maximum pain and away from the center of low back.

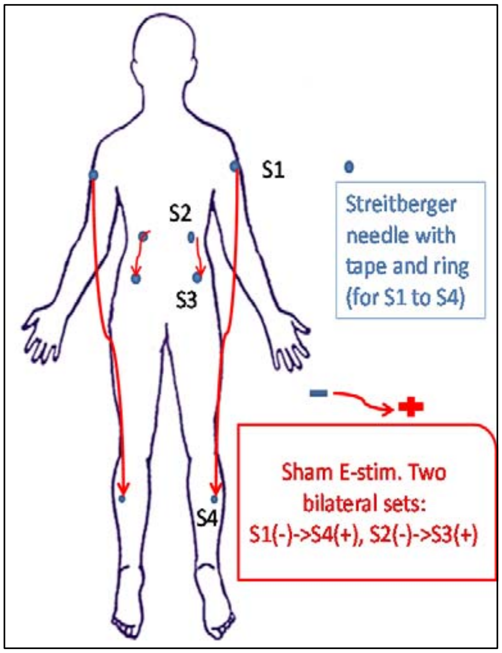

Location of sham points

S1: In the middle of deltoid, 3 Cun below the lateral edge of acromion

S2: 5 Cun lateral to the depression just below T-12 spinous process

S3: 5 Cun lateral to the depression just below L-5 spinous process

S4: Midpoint on the line connecting BL-57 and GB-34 on the posterior calf.

Figure 12- Points Used in the PA Treatment

B. Treatment flexibility

The PA treatment should be identical for ALL participants assigned to the PA arm. There will be no treatment flexibility for PA. We are not concerned that lack of treatment flexibility might unblind the participant as acupuncturists in real life may use fixed points in multiple sessions after a thorough initial evaluation.

C. Sham equipment and procedures to carry out sham stimulation

Equipment: Streitberger needles x 8, with plastic rings + tape to secure their secure placement over the skin. ITO electro acupuncture stimulator x 2 (with intentionally broken wires). Sham needling is achieved by placing non-penetrating Streitberger needles at bilateral S1-S4 sham points. Sham electrical

stimulation is achieved by connecting the Streitberger needles to respective leads on the ITO e-stim machine, with intentionally broken wire, such that no electricity will go through.

No Deqi: During PA administration, the acupuncturist will not attempt Deqi or ask about Deqi while inserting both the Streitberger and the verum needles. Directing the participant's attention to the lack of Deqi on the Streitberger needles will increase the chance of unblinding. Furthermore, none of the needles will be manipulated after insertion throughout the session.

Obviously, the participant may feel different sensations with the Streitberger vs the real needle at GB-21. The different sensations will be pre-emptively addressed by informing the participant that different types of needles (leading to different sensory experiences) will be used for each treatment.

Fake electrical connections: After all needles (sham and verum) are placed, the acupuncturist will connect the sham needles to the fake wires. As shown in Figure 12, the set up will include two circuits carried out by two Ito stimulators. The central circuit (S2, S3) will be connected first, then the peripheral circuit (S1, S4).

Stimulator 1 (central circuit): bilateral S2 to S3 (connect negative lead to S2 on one side and positive lead from the same pair to S3 on the same side, then do the same for the other side using the second pair of leads from the same stimulator).

Stimulator 2 (peripheral circuit): bilateral S1 to S4 (connect negative lead to S1 on one side and positive lead from the same pair to S4 on the same side, then do the same for the other side using the second pair of leads from the same stimulator).

Ritual of intensity titration: Since the stimulator wires are broken, the participant will not feel ANY stimulation. However, we ask the acupuncturist to still turn on the stimulator, adjust the frequency to 2Hz, and bring the intensity dial to 50%. Furthermore, the acupuncturist will carefully perform a mock intensity titration to best mimic the verum scenario, with the following steps:

1) connect the needle handles to the wire leads,
2) inform the participant that the provider will be dialing up the intensity of stimulation,
3) dialing up the stimulator to 50% intensity,
4) inquire of the participant if he/she is comfortable and will be able tolerate the intervention for 20-25 minutes.
5) If the participant questions the lack of sensation, the acupuncturist may reassure the participant that for "this particular type of acupuncture," the stimulation may sometimes be below detection threshold.

D. Additional sham therapies

To best mimic the verum rituals, we will also perform standardized sham heat therapy and sham auricular acupuncture for each PA session.

For the sham heat, we use the same heat lamp, but at a different location (calves), shorter time (10min), and lower intensity (≥1 foot distance). Also in contrast to verum heat which is turned on before needle placement, in sham, we will turn on the heat as the LAST step in treatment (after needles are placed and sham e-stim is titrated).

For sham auricular acupuncture, in order to minimize potential specific effects, we will NOT be using any penetrating needles. Instead, we will be placing two tiny (3mm X 3mm) square tapes at locations specified in Figure 13 below. Neither location is known to have any specific, therapeutic effects. There will be no seeds buried under the tapes, which will be removed at the end of each treatment session.

E. Order of procedures performed in each PA session

The task list and order for a PA session is largely similar to that of a VA session, except for the heat lamp is turned on AFTER all needles are placed in PA and that the protocols involved to place and secure the Streitberger needles are slightly more involved than the verum needle. Furthermore, to re-iterate, in a PA session, NO Deqi sensation will be attempted during all needle placement and no needle manipulation is allowed once the needle is secured. The action orders are summarized below:

Place patient in prone position, place AURICULAR tapes at sham locations.

Place tape and ring on each of the bilateral sham BODY points (S1 to S2, S3, then S4), with minimal palpation of the back. Position and secure Streitberger needles on all eight points.

Connect Streitberger needles to the electrical stimulators and start sham stimulation one stimulator at a time.

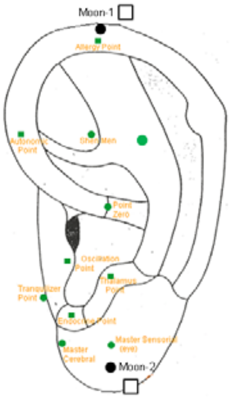

Figure 13 - Sham Auricular Points (Moon1 and Moon 2)

5.2 Handling of Study Interventions

5.2.1 Intervention Accountability Records

The acupuncture interventions will be accounted for in three ways. First, from a materials perspective, about 20-30 needles will be used for each treatment depending which modules are activated by the acupuncturist at the two decision juncture points. Therefore, progress can be tracked by assessing the consumption of needles. Second, from a record-keeping standpoint, the

acupuncturist will record each point she/he needled, as well as level of stimulation by hand and by electrical stimulator in details using a Case Report Form (CRF) which is to be submitted within the same day the treatment is delivered. Third, all treatment sessions will be audio-recorded and the research team will listen to between 1 to 3 randomly selected audio sessions from 20 randomly selected participants.

5.2.2 Insurance of Blinding

This project will involve a parallel arm design including a placebo arm. Similar parallel design of verum electrical and placebo acupuncture treatments were successfully carried out with minimal unblinding by Wayne et al (Arch Phy Med Rehabil Vol 86, Dec 2005). With help from Dr. Rosa Schnyer, a key consultant in the Wayne 2005 trial, we will use a PA design very similar to Wayne as detailed below.

First, Streitberger needles, which are visibly similar to verum needles, will be used (stage-dagger type of device). A thin 1cm diameter plastic ring, secured on skin by paper surgical tape will support the needle and hold it in a perpendicular position.

Second, we will use a standardized script to guide communication between participants and providers for both the verum and placebo arm.

Finally, the effectiveness of blinding will be assessed at the post-treatment assessment visit. We will administer a questionnaire to the participants to ask if they believe they received the more effective or the less effective treatment.

5.3 Concomitant Interventions

5.3.1 Allowed Interventions

Participants may continue their current medication/intervention regimen throughout the project if there are no changes in dosage and the regimen does not include any medications listed in "Prohibited interventions" below. Specific medications that are not exclusionary include:

- Antidepressants
- Antihypertensives
- Inhalers
- Ophthalmic drops
- Thyroid medications
- Opioids < 60mg morphine equivalent units/day

5.3.2 Required Interventions

N/A

5.3.3 Prohibited Interventions

- Benzodiazepines
- Opioid ≥ 60mg daily

- Oral or other systemic steroids
- None of the following interventions are allowed during the trial period
    - Back surgeries
    - Cortisone shots to the back
    - New chiropractic care / maneuvers
    - New physical therapy routines

5.4 Adherence Assessment

5.4.1 Protocol Adherence

Provider's adherence: Based on the geographic distribution of participant in our current P01 study, we found that the majority of our participants are from within 20-mile radius of Palo Alto. The rest are from South Bay (San Jose/Los Gatos), San Francisco and East Bay (Oakland/Berkeley). As such, we decided to hire a main acupuncturist who will be spending two full working days at our facility to provide treatments to the majority of our patients what live around Palo Alto. We hired one acupuncturist from South Bay, SF and East Bay respectively to provide treatments to patients who live far from Stanford/Palo Alto. The names and contact info of the acupuncturists are listed at the beginning of this protocol. The following steps are taken to enhance fidelity of delivery of the interventions.

1. Providers' qualifications: The acupuncturists are licensed and have been in practice for ≥ 3 years. With help from American College of Traditional Chinese Medicine, we were able to advertise our positions to a broad audience of experienced acupuncturists. The providers are competitively selected based on their experience, education, interest and commitment to acupuncture research.

2. Standardization of treatment protocol: Our treatment protocols are standardized for both verum and sham treatments. This will facilitate fidelity and decrease the likelihood of drift over time. Please refer to Provider's Manual for details of the VA and PA treatment protocols.

3. Training of providers:

a. Initial training: each provider will receive 1.5-2 days of training prior to seeing any participant. During day 1, the provider will meet with the PI and the study team, pick up supplies, learn about the study and the rationale behind the treatment protocols, as well as observe and practice the VA and SA protocol at least once. On day 2, the PI will visit the provider's clinic, observe the provider independently perform the VA, PA treatments and independently document the procedures. PI will then provide feedback and answer additional questions. We will schedule Day 2 of the training such that it is within 1-2 weeks of the first participant that the provider is assigned to treat.

b. Regular feedback and support meetings: the PI will review the documentation and audio records of the providers' treatments from 20% of the participants (1-3 randomly selected sessions from 20 randomly selected participants) and provide feedback as issues arise. The feedback will be provided within 1 week of the issue. Additionally, given the challenges of performing clinical trial protocols, PI will hold optional meetings for providers to obtain general feedback and provide support.

4. Adherence monitoring: As suggested by NCCIH, to monitor providers' adherence, we will audio-record ALL sessions from beginning to end. Two aspects of adherence will be monitored, adherence to the general guidelines on communication with participants and adherence with point selection and intensity of stimulation.

a. Fidelity to communication guidelines will be monitored via review of audio-taped sessions. This review will be conducted by the PI and will allow for frequent corrective feedback, should issues with fidelity be detected. The schedule of tape review will be as follows: The initial four sessions from each provider will be reviewed immediately. Subsequently 20% of each acupuncturist's sessions will be reviewed every three months (using the Ceiling function).

b. Fidelity to point selection and stimulation guidelines will be based on review of case report forms. These forms will be filled out by the acupuncturist at the end of each treatment session, electronically and securely, via REDCAP. The schedule of CRF review will be as follows: The CRFs from the initial four sessions of each provider will be reviewed immediately for accuracy, completion, and adherence. Subsequently CRFs from 20% of each acupuncturist's sessions will be reviewed every three months (using the Ceiling function). This review will also be conducted by the PI.

5.4.2 Daily Questions

One secondary objective of the study is to delineate the time to response to electroacupuncture. Though not able to obtain hourly ratings of pain after the treatment, we will be able to collect daily measures of average pain and physical function electronically via REDCAP. The patient will be reminded to fill out this very brief questionnaire (2 questions) daily via a text to their smart phone or an email if the patient does not have access to a smart phone. Additionally, we will collect information weekly via another brief questionnaire on the side effects from the study interventions as well as other treatments obtained by the patient for back pain. This weekly questionnaire will be administered in a similar fashion as the brief daily questionnaire.

5.4.3 Treatment Session Check-in Twice Per Week

Participant attendance at each acupuncture session will be recorded by the acupuncturist and monitored by the PI and the study team.

5.4.4 Measures to Promote Adherence

Measures to promote adherence to treatment protocols by acupuncturists are discussed in Section 5.4.1 above.

Ensuring participant understanding of project expectations

- Knowledgeable and receptive staff: PI and lab managers will train research staff on appropriate languages for consenting as well as setting appropriate expectations for study participants.
- Provide a Welcome Packet with project information and easily found contact information for project staff.

- Develop a personal relationship with the participant
- Continued outreach: After enrollment, PI will write letter to participant thanking him/her for their work/participation and outline/remind participant of study timeline. Birthday and holiday cards may also be sent to the participant periodically.

5.4.5 Specific Triggers and Stopping Rules

All analyses performed in this project will be intent-to-treat.

If participants miss more than 3 treatment sessions, their participation will be considered incomplete. Out of the 3 missed sessions allowed, only two may be consecutive. All non-completers will have the opportunity to resume treatment even if their data is considered incomplete.

Post-treatment data will be collected on non-completers identically as on the completers. In addition to ITT, we will also run a per-protocol analysis where the non-completers will not be included.

6. STUDY PROCEDURES

6.1 Schedule of Evaluations (see next page)

Who Completes	Assessment	Screening Visit	Baseline Tracking	Pre-Tx Visit	Tx #1	Tx #2	Tx 3,4, 6-11	Tx #5	Tx #12	Poxt-Tx Visit	Follow up 3,6,12 mo
**Participant**	Informed Consent	X									
	Demographics	X									
	Pain Intensity (30 d) (NRS)	X									X
	Pain Intensity (7 d) (NRS) – to be added			X						X	
	BodyMap	X		X						X	X
	NIH Dataset	X									
	STartBAck Tool	X									
	Medical History	X									
	Medication List	X									
	Questionnaire Packet #1	X									
	Daily Symptom Tracking		X	X	X	X	X	X	X	X	
	Weekly AE, BodyMap, Med tracking		X	X	X	X	X	X	X	X	
	RMDQ			X						X	X
	PROMIS Packet			X						X	
	Questionnaire Packet #2			X						X	
	Expectation				X						
	Vincent Credibility					X					
	Blinding Assessment								X		
Acupuncturist	Acupuncturist Intake Form				X						
	Tx Record				X	X	X	X	X		
	Adverse Events				X	X	X	X	X		
	treatment appraisal questionnaire							X			
	Session 5 Form							X			
**Research Team**	PH-9	X									
	Incl/Excl CRF	X									
	IC CRF	X									
	Physical Exam	X									
	QST	X		X						X	
	Vitals/HRV	X		X						X	
	Screening Visit CRF	X									
	Pre-Tx Visit CRF			X							
	Enrollment	X									
	Randomization			X							
	Post-Tx Visit CRF									X	
	Completion CRF									X	
	Protocol deviation log									X	
	Cumulative AE log									X	
	Monthly QA CRF	Monthly on consents, all data entry, timing compliance / protocol deviations, AE's and audio tapes									

Comment [p1]: Please note that all weekly surveys are identical. We start administering them at the completion of the baseline visit and continue until the completion of the post-treatment visit. What is shown in this table represents a typical timeline. The total number of weekly questionnaires administered may vary due to the difference in the time lag between the assessment visits and the start and end of the treatment period.

Questionnaire Packet 1: Fear-avoidance behavior questionnaire (FABQ), coping skills questionnaire-revised (CSQ-R), PTSD checklist-civilian, PTSD history questionnaire,

Questionnaire packet 2: pain catastrophizing scale (PCS), illness perception questionnaire (IPQ), , caffeine intake questionnaire, pain self-efficacy questionnaire (P-SEQ)

PROMIS packet (CAT version): pain intensity, pain interference, physical function, depression, anxiety, anger, sleep related impairment, sleep quality, fatigue.

6.2 Description of Evaluations

Refer to Appendix A for schedule of events.

6.2.1 Screening Visit and Enrollment

These evaluations occur to determine if the candidate is eligible for the project, and to collect initial assessments for the study after the participant is determined eligible and consented.

Pre- Screening

Initial eligibility will be assessed via an online platform with basic eligibility questions. Further eligibility will be assessed via a telephone screen based on the inclusion/exclusion criteria. Individuals who meet preliminary criteria will be scheduled for their Screening Visit.

The Screening Visit

The screening process may last 1-4 weeks from the time the participant is screened over the phone to the time of enrollment (ie screening visit) depending on the mutual availability of the participant and the research team. On this visit, informed consent will be obtained, followed by eligibility determination, enrollment, additional evaluations and scheduling of the subsequent, baseline visit.

Informed Consent

At the beginning of the screening visit, a member of the research team will consent the patient for the procedures of the study (evaluations, treatment, timeline, follow up, HIPAA) etc. For details on the consenting process, please refer to section 4.3.3. All participants will be provided with a Welcome Packet which may include: copies of their consent forms, brochures on the research project, study appointment information, a timeline of participation, and relevant contact numbers.

Assessing for Eligibility

After the informed consent is obtained, the eligibility assessment will include the following evaluations:

- Mean Pain Intensity Over 30 days
- Medical History questionnaire
- Prior and Concomitant Medications
- Demographics
- Brief physical exam
- PH-9 (patient health questionnaire)
- Confirmation of meeting inclusion/exclusion criteria

Eligibility Determination and Enrollment

Eligibility determination: The patient will undergo the evaluations above and the research team will check the patient's eligibility against an inclusion/exclusion checklist. Once the patient passes the checklist, he/she will be deemed eligible for the study and will be enrolled (see below). If the patient is

not eligible, we will ask his/her permission to be included in the database so we can contact him/her for future studies which may be suitable.
Enrollment procedure: Once the patient is deemed eligible, he/she will be enrolled for the project and assigned a project ID. The enrollment date will be the date of the screening visit and will be recorded in the SNAPL Central Database. Enrollment date and future visit dates will also be recorded in the project database on REDCAP for tracking purposes.

Additional evaluations

- **Questionnaires:** key assessment tools of chronic low back pain (NIH minimum dataset, Start Back Tool, BodyMap) will be collected at the visit. For the sake of time, a packet of additional questionnaires (#1) will be emailed to the participant who will be reminded to fill these out during the subsequent week.
- **Additional Physical Assessments:** In addition to the physical exam (which was part of the screening procedure) we will collect data on QST and vital signs, including measures of heart rate variability [HRV].

Daily and weekly symptom tracking: Throughout the study, starting at enrollment, the patient will be emailed a brief set of **daily** questions assessing his/her back pain and related symptoms. The importance of answering these questions will be stressed with the patient. In addition, a **weekly** survey will be emailed to each participant, assessing for presence of adverse event, locations of pain via the BodyMap, and any new treatments (meds or procedures) started for back pain. All weekly surveys are identical. We start administering them at the completion of the baseline visit and continue until the completion of the post-treatment visit. What is shown in the table above represents a typical timeline. The total number of weekly questionnaires administered to each individual may vary due to the difference in the time lag between the assessment visits and the start and end of the treatment period. If a participant misses >50% of the weekly surveys, it will be considered a protocol deviation.

6.2.2 Pre-treatment visit and Randomization

One to 2 weeks after the screening visit, the participant returns to SNAPL for a baseline (aka pre-treatment) visit.

Pre-treatment/Baseline Assessments

- **Questionnaires:**
  - A second set of **questionnaires (packet #2),** known to be highly predictive of response to acupuncture, will be administered to the participant during the baseline visit. These questionnaires include: pain catastrophizing scale (PCS), illness perception questionnaire (IPQ), treatment appraisal questionnaire, caffeine intake questionnaire, pain self-efficacy questionnaire (P-SEQ).
  - State-measures assessing back pain and associated symptoms via the **NIH PROMIS instruments** (depression, anxiety, pain intensity, physical function, pain interference, sleep quality, sleep-related impairment, fatigue, anger) will be administered. To minimize participant burden, all PROMIS instruments will be administered in the computer adaptive (CAT) format, which should not take 4-6 questions per instrument.

- **Physical Assessment:** Data will be collected on **vitals**, **HRV**, as well as a select set of **QST** measures (should be shorter than the initial, screening visit as no optimization steps will need to be repeated). We will NOT be repeating the physical assessment on the baseline visit.
- **Checking on completion of daily and weekly questionnaires:** While the completion of the daily (and weekly) questionnaires are no longer required for enrollment into the study, it is still important that participants fill out these questionnaires to provide accurate data on baseline symptoms as well as during and immediately after the study intervention. We will check the completion of the questionnaires. If a significant portion is not filled out (>50%) we will inquire the reasons for the lack of completion and work with each participant to improve the completion rate (identify barriers, consider reminders via phone 1-2x per week etc). We will also document any participants that complete less than 50% of their daily questionnaires as a protocol deviation.

Randomization

Randomization to a specific treatment arm will take place after the baseline visit, before the first treatment session. The study intervention will begin as soon as possible (within 1 week ideally) after the Baseline Visit. The randomization procedure is detailed below:

- ☐ Participant becomes ready for treatment after completion of baseline visit
- ☐ Research team assign participant to an acupuncturist based on participant's location preference.
- ☐ Research team provide participant the acupuncturist's contact info and the acupuncturist participant's contact info.
- ☐ Acupuncturist and participant reach out to each other and schedule first treatment appointment.
- ☐ Once the appointment is made, acupuncturist alert research team, who then alert an outside party (another RC from or lab not related to our project) who then performs randomization by entering the participant ID and acupuncturist ID into the randomization program.
- ☐ The randomization program is written in R, and we will stratify randomization per acupuncturist with a block of 4 within each acupuncturist, to ensure balanced treatment assignment within each provider.
- ☐ The unrelated RC will then securely email the treatment assignment (participant ID and treatment arm) to the acupuncturist.

6.2.3 Blinding

<u>Blinding of the participant</u> will be ensured by strict controls in the communications between the acupuncturist and the participant. The acupuncturists will receive one day of hands-on training as well as written materials, and they will attend a refresher workshop 1-2 weeks before seeing their first participants. Strict guidelines on communication with the participant will be provided and will be reinforced during regular provider meetings with the PI (JT Kong).

<u>Blinding of the assessor.</u> The PI and the research assistant will be the assessors. Only the acupuncturists (treatment providers) will be unblinded. The randomizer will deliver the randomization assignment to the acupuncture provider via secure email.

Individuals authorized to break the blind. The members from the DSMB will meet and assess side effects from both treatment arms and decide if there is a need to unblind and to possibly terminate or modify the problematic treatment.

Circumstances for breaking the blind: We do not anticipate that we will need to break the blind unless we see clear harm (>50% worse from baseline for one group, or >30% participants experience prolonged side effects, e.g. severe pain, persistent hematoma, nausea etc) lasting over two weeks.

Procedures for breaking the blind. We stress that we do not anticipate breaking the blind except for extenuating circumstances where significant harm is caused by the VA or PA treatment. Such circumstances will always be reported to the DSMB and a decision regarding how to proceed will come from the DSMB. Such examples include a) large bleeding and bruising (over three sessions) due possibly to an undiagnosed clotting disorder; b) severe pain due to the treatment as recorded on the participants weekly adverse event report form lasting more than two weeks; c) unresolved nausea, dizziness persistent for more than two weeks.

In terms of procedures for breaking the blind, we will use the following steps.

Level 1: The research assistant will be responsible for monitoring the participant's adverse event data gathered via the Android device; additionally, the acupuncturist will monitor and report on side effects from treatment. If any of the above mentioned severe reactions to acupuncture occur (extremely rare), the RC and/or the acupuncturist will report to the PI.

Level 2: The PI will report the event(s) to the DSMB.

Level 3: The DSMB will decide, based on the severity and time course of the reported adverse, whether or not to terminate the participation of the participant and at the same time to break the blind. The information of treatment delivered and settings where the adverse event occurred would be important to minimize such events from happening again in the future.

6.2.4 Treatment Visits

Treatment Visits. Each participant will receive 12 treatment sessions. For each session, the participant fills out adverse event form at the beginning of the session (except for the first session), the acupuncturist fills out the treatment CRF near the end of the session. Sessions 1, 2, 5 and 12 will contain specific assessments, as outlined below.

Treatment Session 1: 60 minutes

- Acupuncturist will perform initial history and physical. History will include similar information as original baseline visit (pain location, duration, character, intensity, medication list, past medical and surgical history, previous allopathic and CAM treatment for back pain; as well as at TCM related question. Physical exam will include pulses, tongue exam according to TCM, as well as lumbar and low extremity palpation, range of motion, as well as anything else can be comfortably performed by the acupuncturist. These details will be recorded in the **Acupuncture Intake CRF**.
- Most importantly, the acupuncturist will record a) if the participant experiences pain in hips/upper thighs; b) rating of back pain over the last 7 days using an 11-point (0-10) numerical rating scale (NRS).

- Treatment will be administered per protocol (see Section 5.1)
- Participant will complete **Stanford Expectancy of Treatment** BEFORE and AFTER the 1st treatment
- Acupuncturist will complete session record on Visit Notes CRF
- Acupuncturist will schedule/confirm next treatment session(s)

Treatment sessions 2: 45 minutes

- Participant will complete adverse event form before session starts.
- Treatment will be administered per protocol (see Section 5.1)
- Participant will complete **Vincent Credibility Scale** after the 2nd session.
- Acupuncturist will complete session record on Visit Notes CRF
- Acupuncturist will schedule/confirm next treatment session(s)

Treatment session 5: 45 minutes

- Participant will complete adverse event form before session starts
- Acupuncturist will fill out pretreatment **assessment of reduction in pain**, and determine if additional treatment module will be used for this and upcoming sessions in the verum arm (sham arm does not change protocol but will perform identical assessment). Refer to 5.1.2.B and 5.1.3.B for details.
- Treatment will be administered per protocol (see Section 5.1)
- Acupuncturist will complete session record on Visit Notes CRF
- Acupuncturist will schedule/confirm next treatment session(s)

Treatment sessions 3, 4, 6-11: 45 minutes

- Participant will complete adverse event form before session starts
- Treatment will be administered per protocol (see Section 5.1)
- Acupuncturist will complete session record on Visit Notes CRF
- Acupuncturist will schedule/confirm next treatment session(s)

Treatment sessions 12: 45 minutes

- Participant will complete adverse event form before session starts.
- Treatment will be administered per protocol (see Section 5.1)
- Participant will complete the simple **blinding assessment questionnaire** at the end of the treatment.
- Acupuncturist will complete session record on Visit Notes CRF

6.2.5 Post-Treatment Visits & Follow-up

Post-Treatment Follow-Up Visit. The Post-Treatment Visit will be conducted approximately 1-2 weeks after the last (12th) treatment session. Similar assessments to the baseline visits will be performed.

- **Questionnaires:** All questionnaires will be administered via REDCap. See Table 1 for details.

- **Physical Assessment:** We will record vital signs and HRV of the participants.
- **Quantitative Sensory Testing (QST):** Participants will undergo QST. See Appendix 2 – QST Manual for complete description of tests, devices, and safety parameters.

Online Follow-Up. Participants will complete online assessments at 3, and 6 and 12 months post-treatment. These assessments will include a limited dataset out of the questionnaires that the participant completed at their Baseline Visit (RMDQ and mean pain intensity). Specifically, these questionnaires are scheduled to reach the participant at 3, 6, and 12 months measured from the LAST day of intervention. Our automatic survey invitation is programmed such that, if the participant has not responded, the questionnaire will be sent 3 more times weekly until a response is entered.

In order to increase the capture of long-range data, the research team will also reach out via email or phone to the participants who have missed all 4 email reminders. The windows for completions are: up to 6 months for the 12-month survey, up to 3 months for the 6-month survey and up to 1 month for the 3 month survey. In order to find the balance between maximizing data capture and accuracy of recall, for the participants who are outside their window, we ask them to recall the approximate average back pain intensity at 3, 6 or 12 months after their last study intervention.

Debriefing. On the post-treatment assessment visit, all participants will be debriefed by RC the research team. The PI or the RC will only unblind the participant AFTER all assessments are completed at the post-treatment visit.

The table below summarizes the visits for the project.

Week Number*	Visit	Events
Week -1	Screening Visit	Informed consent, eligibility determination/enrollment, questionnaires, physical exam, HRV/QST, daily and weekly tracking
Week 0	Pre-treatment/baseline assessment	More questionnaires, HRV/QST, randomization
Week 1	Treatment sessions 1 and 2	Treatment per protocol
Week 2	Treatment sessions 3 and 4	Treatment per protocol
Week 3	Treatment sessions 5 and 6	Treatment per protocol
Week 4	Treatment sessions 7 and 8	Treatment per protocol
Week 5	Treatment sessions 9 and 10	Treatment per protocol
Week 6	Treatment sessions 11 and 12	Treatment per protocol
Week 8	Post-Treatment Follow-Up Visit	Questionnaires, QST, HRV, physical exam, Debriefing.
~ Week 18	Online/Telephone Follow-up 1	Questionnaires at 3 months post-treatment
~ Week 32	Online/Telephone Follow-up 2	Questionnaires at 6 months post-treatment
~ Week 58	Online/Telephone Follow-up 3	Questionnaires at 12 months post-treatment

*This table assumes the most ideal situation: 1 week between screening and baseline and beween baseline visit and treatment initiation, and 1-2 week period between end of intervention and post-treatment visit.

6.2.6 Study Completion

Study Stop Point CRF

Research staff will complete the first half of the study stop point CRF indicating the completion of the participant's study visits. There will be a separate section on the Study Stop Point CRF, which will be completed after the participant completes their last online follow-up. At this point, it will be indicated on the CRF that all follow-up visits are complete and the participant's record will be closed.

Handling of Early Termination:

- List of possible reasons: scheduling conflicts, intolerable adverse events, no longer interested, lack of benefits (analgesia), pregnancy, development of significant medical/psychiatric comorbidity or additional pain foci.
- Necessary evaluations: Most importantly, we will collect the daily monitoring data and use the average pain score from the last 7 days prior to termination to calculate the final primary outcome (pain reduction). Depending on the severity of side effects, we will bring the participant in for a visit with one of our MD staff, provide appropriate treatments, and monitor the participant until the side effects resolve. For details of the type of side effects and prospective treatments, please refer to Section 7.3 under "adverse effects."

7. SAFETY ASSESSMENTS

Overall, acupuncture is a generally safe procedure with essentially negligible risk for serious complications in practiced hands (which is true in our case as all our acupuncturist have >3 years of practice experience).

Potential adverse events: acupuncture is an extremely safe intervention. For each of the **four categories of complications**, we have specific ways to handling them as below.

- **First**, the most common side effects are minor pain and bruising at the needle insertion sites and they typically go away within 2-3 days.
- **Second**, some individuals may experience mild vasovagal reaction near the beginning of the treatment. If it happens, we will immediately lay the participant supine and monitor. Only if these symptoms do not resolve after the participant is laid supine for 5 minutes, we plan to vigorously stimulate Du-26 and select Ting points at the toes which are indicated to treat vasovagal reactions. If within 2 minutes the symptoms do not subside, we will take the needles out, and offer hydration. Most cases resolve with these maneuvers.
- **Third**, nausea and dizziness may also occasionally occur but are short-lived. If they do occur, we will immediately lay the participant supine and monitor. Only if these symptoms do not resolve after the participant is laid supine for 5 minutes, we will insert needles to bilateral MH-6, ST-41 (indicated to treat nausea), and GV-20 (indicated for dizziness) in the subsequent sessions. If the symptoms do not improve after 3 sessions and the participant feels significantly bothered, we will terminate the participant's enrollment.
- **Fourth**, complications such as infection, nerve injury and pneumothorax are extremely rare (less than one in 10,000). If they do occur, treatment will be terminated, DSMB will be alerted, and the participants will be followed and treated by an MD until the symptoms resolve following

standard of care (e.g. antibiotics for infection, x-rays and possibly needle decompression for pneumothorax).

- **Finally**, adverse reactions will be recorded two ways: by the acupuncturist at the end of each session and by the participant on their Android device.

7.1 Specification of Safety Parameters

The most common risks in acupuncture are local swelling, bruising, and pain, and less commonly transient nausea and dizziness. In the rare case of persistent light-headedness, blood pressure and heart rate will be assessed in the office. Most of the monitoring will be done by verbal report by the participant and visual exam and palpation of the needled sites by the acupuncturist.

7.2 Methods and Timing for Assessing, Recording, and Analyzing Safety Parameters

The surveillance and detection of adverse effects by acupuncture will be accomplished in three layers.

Layer 1 – the participant: The acupuncturist will explain the common side effects of acupuncture to the participant based on a standardized script. Then, the participant will log the presence of any side effects on the Android device on a weekly basis. Further, presence of immediate side effects to treatment will be reported to and noted by the acupuncturist on the session record.

Layer 2 – The acupuncturist: he/she will observe and inquire about immediate side effects after each treatment, will also note delayed side effects on the session records.

Layer 3 – The research team (PI and RC) will survey the Android device logs by the participants every 2-4 weeks and review the presence, time course, and severity of side effects, and intervene if necessary.

7.3 Adverse Events and Serious Adverse Events

An **adverse event (AE)** is generally defined as any unfavorable and unintended diagnosis, symptom, sign, syndrome or disease which either occurs during the project, having been absent at baseline, or if present at baseline, appears to worsen. Adverse events are to be recording regardless of their relationship to the project intervention.

A **serious adverse event (SAE)** is generally defined as any untoward medical occurrence that results in death, is life threatening, requires inpatient hospitalization or prolongation of existing hospitalization, results in persistent or significant disability/incapacity, or is a congenital anomaly.

The participants will be provided with emergency phone numbers to contact the team in case of serious side effects.

At each treatment visit, the acupuncturists will record all adverse events in the Adverse Events CRF and will notify the Clinical Core.

The daily questions completed by participants on Androids will capture participant mood as well as common side effects. Triggers will be programmed into the application for a negative mood rating, which will alert the Clinical Core after 5 consecutive reports of greater than 30% negative change in mood as compared to baseline. The Clinical Core will then follow-up with the participant by phone to inquire about the negative mood rating and record it in the Adverse Event CRF.

Finally, there will be a short questionnaire programmed into the Android device to ask about side effects and adverse events at the end of each week. The Clinical Core will monitor these weekly reports and

follow-up with the participant by phone if side effects are reported and an Adverse Event CRF will be completed for the participant's record.

7.4 Reporting Procedures

Note that adverse events are recorded in 1) participant weekly surveys; 2) acupuncturist's CRF's; 3) at post-treatment visit by research team. The research team will perform QA check on all of the following data (with particularly focus on safety) on a monthly basis: participant daily/weekly surveys, participant questionnaires, consent forms, all research team CRF's, acupuncturist's CRF's. Specifically, we will check for 1) protocol deviations; 2) all adverse events. We will log these for each participant on Excel Spreadsheets (on our lab's HIPPA compliant server), monthly and report all to NCCIH, DSMB and IRB at the appropriate time intervals.

Acupuncturists will assess adverse events twice a week at the treatment sessions and will complete the Adverse Event CRF. Acupuncturists will also notify the RC in case of adverse events and the RC will follow-up with the participant by phone within 3 business days if the adverse events are acupuncture treatment related. If adverse events are severe and related to the acupuncture project, DSMB will be notified immediately, the treatment will be stopped and the PI will have a consultation with the participant within 3 days of the onset of side effects.

Adverse Events will be reported to NCCIH, DSMB, and Stanford IRB annually. Serious Adverse Events that are determined to be related to the study will be reported to the IRB by filing a report on the Stanford IRB website. A copy of this report will be sent to the NCCIH officer. Unexpected fatal or life-threatening Adverse Events related to the intervention will be reported to the NCCIH Program Officer within 7 days. Other serious, unexpected, and related Adverse Events will be reported to the NCCIH Program Official within 15 days and to the Stanford IRB within 10 business days. Anticipated or unrelated Serious Adverse Events will be handled in a less urgent manner but will be reported to the Independent Monitor(s), Stanford IRB, and NCCIH in accordance with their requirements. In the annual Adverse Event summary, the Independent Monitor(s) Report will state that they have reviewed all Adverse Event reports.

7.5 Follow-up for Adverse Events

After Adverse Events are discussed by the RA/PI and participant and a decision is made regarding continuation of participation, the RC will conduct a 1-month follow-up phone call to the participant. This phone call will also be recorded in the Adverse Event CRF.

7.6 Safety Monitoring

Data Monitoring and Safety Board (DSMB)

The CAM Center DSMB has the following members:

Dr. Manisha Desai, PhD (Biostatistics expertise)

Dr. Karen Sherman, PhD (TCM trials expertise)

Dr. Remy Coeytaux, MD/PhD (Clinical expertise)

The DSMB will convene every 3 to 6 months after initial recruitment. Safety will be reviewed and participant cases will be reported to the members of the DSMB and relevant safety decisions will be

made. The sessions will be open to PI and the mentors. DSMB reports will be sent to NCCIH and Stanford IRB within 10 business days of the meeting.

8. INTERVENTION DISCONTINUATION

Participants will be free to withdraw from the project if they no longer wish to participate. If participants do not attend more than 3 sessions of PA or VA project arms, their participation will be considered incomplete. However, they will not be withdrawn from the project and will be given the opportunity to resume treatments. Since this project is not monitoring any lab values and does not involve drugs, there are no such values that would warrant intervention discontinuation. As mentioned above, participant mood rating and side effects will be monitored via the Android devices, and participants will be withdrawn from the project by the Investigator if it is determined that the intervention is causing the negative mood ratings, persistent worsening pain, and/or side effects that cannot be resolved.

9. STATISTICAL CONSIDERATIONS

9.1 General Design Issues

This is a randomized control trial in which 100 adult participants with chronic low back pain (CLBP) will be randomized to 6 weeks of twice a week sessions with verum acupuncture (VA) or placebo acupuncture (PA). Participants and researchers will be blind to treatment assignment. Treatment providers will not (and cannot) be masked to treatment assignment

Aim 1. Explore the predictive relationship and the association between **ascending sensitization** measures and clinical response to acupuncture.

Hypothesis 1: augmented temporal summation (TS), and more wide-spread pain (WSP) (quantified by number of painful areas marked by the participant on a standardized body map) will be associated with greater pain reduction in the verum arm than in the placebo arm; decreased pressure pain threshold (PPTr) will be associated with greater pain reduction in the placebo arm than the verum arm.

Aim 2. Explore the predictive relationship and the association between **descending modulatory** measures and clinical response to acupuncture.

Hypothesis 2: decreased conditioned pain modulation (CPM), and more favorable psychological state, measured by less pain catastrophizing, better self-efficacy in coping and in pain management, will be associated with greater pain reduction in the verum than the placebo arm; expectation of positive treatment outcome will be associated with pain reduction in both the verum and the placebo arm.

Aim 3. **Exploratory model building** to predict pain reduction by electroacupuncture.

Hypothesis 3. A mathematical model relating key baseline characteristics to percent pain reduction will be developed using advanced techniques, and this model will be tested using cross-validation within the proposed pilot.

9.2 Sample Size and Randomization

Participants

The project will enroll 100 adults (ages 21-65 years) who meet criteria for chronic axial low back pain without radicular symptoms. Participants with CLBP will be randomly assigned to an equal duration of acupuncture treatment or placebo.

Power Analysis

The objectives of aim 1 are to 1) explore the predictive relationship between ascending factors of interest and pain reduction due to our interventions; 2) test the association between the ascending factors and pain reduction. However, because the former is exploratory in nature, we will design our study to power for the latter objective. Specifically, we compute the number of patients needed to find an association between key baseline parameters (pressure pain threshold, temporal summation (TS), or widespread pain) and pain reduction due to verum treatment. With 50 patients per arm, we will be able to detect a moderate correlation of 0.4 (supported by literature for pressure pain threshold11 and TS67) with approximately 80% power with alpha of 0.05 using Fisher's transformation. Next, to determine if the association is stronger in the verum than the sham arm, we will test the interaction between treatment and the baseline parameters. Fifty patients per arm will provide 80% power (with alpha of 0.05) to detect a moderate difference of 0.5 between correlation from the verum arm and that from the sham arm. Therefore, we are adequately powered to detect a moderate association between baseline parameters and the clinical response in the verum acupuncture arm and a moderate difference in the strengths of these associations between the verum and the sham treatment group.

Similar calculation with identical numbers can be found for aim 2 (descending factors). Note prior literature supports a moderate association around 0.4 between catastrophizing and pain reduction as well as between self-efficacy and pain reduction in patients suffering from chronic low back pain.

Randomization

When a new participant has passed the initial screening and baseline visit, he/she will be randomized to receiving either VA or PA with equal chance. An R program for variable block randomization will be used. We will randomize patients in blocks with block sizes randomly selected from 6, 8 and 10. Our lab manager or his associates (who are NOT immediately related to the proposed project) will run the randomization program and inform the treatment assignments to each provider via secure emails. Note that the participant will be able to pick the acupuncturist based on ease of access (commute time).

Details of Blinding can be found in Section 6.2.3 Blinding. Details on randomization can be found in Section 4.3.2.

9.3 Definition of Populations

ITT (Intent to treat) refers to all participants who have completed the baseline assessments and have been randomized to 1 of 2 arms.

Modified ITT refers to all participants who have attended at least one session and completed the measure at the screening and baseline visits.

Per treatment refers to participants who have completed (no more than 3 missed sessions) either VA or PA and the assessments (both baseline and post-treatment visits).

9.4 Interim Analyses and Stopping Rules

The most common risks in acupuncture are local swelling, bruising, and pain, and less commonly transient nausea and dizziness.

We do not anticipate that safety reviews will be necessary unless we see clear harm (>50% worse from baseline for one group, or >50% participants experience prolonged (>2 weeks) moderate or severe side effects (eg nerve injury, large hematoma with diameter > 5cm, or increased back symptoms that prevent

the participant from tending to normal work and recreational activities which were not a problem prior to participating in the study). Such findings will be reported to the PI and the lab manager to review the events by group to determine whether there are statistical as well as clinical concerns. The research team will report the findings to NCCIH. The findings are used to determine what steps will be taken, such as to determine whether the project should continue per protocol, proceed with caution, be further investigated, be discontinued, or be modified and then proceed.

9.5 Outcomes

9.5.1 Primary Outcome

Primary outcome: Change in Pain Intensity measured by the T-scores computed from the full PROMIS Pain Intensity Instrument administered at the pre-treatment and post-treatment visit.

9.5.2 Secondary Outcomes

Secondary outcomes: a) percent pain reduction at 6 and 12 months from baseline; b) percent change in physical function, measured by RMDQ or the pain interference instrument from PROMIS at the post-treatment visit, and at 3, 6 and 12months from baseline. C) percent change in pain intensity measured by the 11-point NRS at both the pre- and post- treatment visit.

9.6 Data Analyses

Aim 1. Explore the predictive relationship and the association between ascending sensitization measures and clinical response to acupuncture.

Planned analysis: There are two specific goals of the analysis: (1) explore the predictive relationship between key baseline parameters and treatment response (pain reduction) accordingly by arm; and (2) examine the associations between baseline predictors and treatment response by arm. The identified association can help us understand the underlying mechanism of the treatment effect from acupuncture and, AND assist in building good subsequent prediction models. To this end, taking temporal summation (TS) first as an example, we will first estimate the correlation coefficients between baseline TS and the primary outcome (% pain reduction immediately after treatment) and then perform multivariate linear regression with clinical pain reduction as the continuous dependent variable and TS and potential confounding factors such as the demographic variables and the baseline pain level as independent variables in the verum arm. We will estimate the regression coefficients and their 95% confidence intervals, which will be used to formally test association between TS and pain reduction with or without adjustment of potential confounders. To evaluate the predictiveness of TS (with other patients' characteristics) to pain reduction, we will also calculate the mean squared prediction error of the multivariate regression model. Variable selection method such as lasso may be used to remove unnecessary predictors from the model and cross-validation will be used to estimate the prediction accuracy to account for the adaptive model building step. Likewise, we will repeat the analysis in the sham arm. Specifically, we will test the presence of the association by examining if zero is inside the interval for confirming the hypothesis that the association is close to null in the sham arm. We will also estimate the interaction between the treatment group and TS to determine if decreased TS better predicts treatment response to verum than to sham acupuncture. Note that factors which are equally predictive to the pain reduction in both arm in general do not have interaction with the treatment.

In summary, the primary objective will be to assess the predictiveness of baseline TS to pain reduction; and the primary outcome of pain reduction will be the difference in pain between baseline and immediately after treatments. The primary analyses will be the computation of mean-squared prediction error from a multivariate regression model, as well as the use of Lasso and cross-validation to improve and validate the predictive model with TS. The second objective will be to quantify the association between TS and pain reduction immediately after treatments (ie secondary outcome is the same as the primary outcome, pain reduction immediately after treatments). The secondary analyses will be a) computation of the correlation coefficient between baseline TS and pain reduction and b) estimation of the regression coefficient and 95% confidence intervals between baseline TS and pain reduction in a multivariate regression model taking TS and other baseline variables into account.

The same analyses will be applied to pressure pain threshold and widespread pain. Realizing that pain reduction may not be normally distributed and that the assumed linear regression model may not be appropriate, we will repeat the above analysis using a conservative, semi-parametric transformation model. We will perform the parallel logistic analysis with dichotomized pain-reduction (responder vs non-responder) as the binary dependent variable of interest. We will perform similar analyses for widespread pain. Finally, we will repeat the above using pain reduction at 6 months post-treatment as our outcome variable.

Aim 2. Explore the predictive relationship and the association between descending pain modulatory measures and clinical response to acupuncture.

Planned analysis: the statistical plan for Aim 2 is almost identical to that of Aim 1 except, we replace baseline factors of TS, pressure pain threshold and widespread pain, with baseline CPM, pain castrophizing, coping effectiveness and self-efficacy. In summary, the primary objective will be to assess the predictiveness of baseline descending pain modulatory factors (CPM, pain catastrophizing, coping effectiveness and self-efficacy for pain management) to pain reduction; and the primary outcome of pain reduction will be the difference in pain between baseline and immediately after treatments. The primary analyses will be the computation of mean-squared prediction error from a multivariate regression model, as well as the use of Lasso and cross-validation to improve and validate the predictive model with each descending pain-modulatory factor of interest. The second objective will be to quantify the association between each descending pain-modulatory factor of interest and pain reduction immediately after treatments (ie secondary outcome is the same as the primary outcome, pain reduction immediately after treatments). The secondary analyses will be a) computation of the correlation coefficient between each baseline descending factor and pain reduction and b) estimation of the regression coefficient and 95% confidence intervals for between each baseline descending factor of interest and pain reduction in a multivariate regression model.

Finally, recognizing the exploratory nature of our study aims, we appreciate the fact any significant findings from the analyses from both Aim 1 and Aim2 may be due to artifacts of multiple looks at the data. Therefore, we will a) consider correction for multiple comparisons in our final analysis; b) verify the significant findings from our pilot exploratory study in subsequent, larger, controlled clinical trials.

Aim 3. Exploratory model building. I will explore mathematical models relating acupuncture analgesia to baseline characteristics, and test this model within the proposed pilot via cross-validation.

Planned analysis: Model-building. We will train a predictive model based on all factors examined in Aims 1 and 2, and additional baseline measures including demographics, job status, history of childhood trauma (PTSD), fear avoidance beliefs, and current symptoms of mood disorders. We will develop multivariate prediction models for predicting pain-reduction after treatment. Lasso-penalized logistic/linear regression, regression tree, random forest, boosted CART, and other data mining techniques will be employed. Using cross-validation, we will check the quality of our models by mean-squared prediction error for continuous outcome; and by misclassification error and sensitivity/specificity in the ROC curves for dichotomized outcomes. The prediction model will be trained in the verum and sham arms separately. In the end, we will balance simplicity, interpretability and the prediction performance to select the optimal model, where the prediction performance will be objectively evaluated again with cross-validation. The final constructed models can be used for guiding and developing personalized delivery of acupuncture.

Validation. We will test the performance of the selected prediction model on patients via cross-validation by comparing the observed and predicted outcome. The performance will be evaluated via metrics, including mean-squared error, misclassification rate, sensitivity, specificity, and ROC curve, when appropriate. Importantly we will predict the treatment benefit of each patient using the chosen models and will identify the subgroup of patients with the highest predicted benefit of verum relative to sham. Then we will estimate the treatment effect in the identified subgroup of patients. If the actual pain reduction in verum arm is significantly higher than sham among the selected subgroup of patients, then the selected model will be deemed successful and may be used for recommending acupuncture in clinical practice.

10. DATA COLLECTION AND QUALITY ASSURANCE

10.1 Data Collection Forms

All questionnaires will be completed by participants in the REDCap Database. If some questionnaires have to be collected on paper due to unforeseen circumstances, the forms will be stored as source data and a member of the research team will enter the data into the REDCap database.

Participants will complete majority of the questionnaires associated with the pre- and post-treatment visits on-site. To ease participant's burden during their onsite visit, we will send longer questionnaires and those with relatively less predictive power based on prior studies via weblinks such that participants can complete them at the comfort of their home. All follow-up questionnaires will be completed online by the participants at home.

The research team will fill out the majority of the CRF's and keep these along with the Phone Screen as source data. The acupuncturists will fill out a CRF for initial intake and a CRF for each acupuncture session to record details of the treatment.

All participants will be identified by a unique project ID number on their corresponding data and case report forms and will not be identified by their name. All CRFs will be stored in binders identified by the Participant ID number and stored in locked cabinets. All staff will receive training on completing CRFs appropriately, reviewing CRFs for completeness, and maintaining participant confidentiality.

10.2 Data Management

The PI and the RC will be responsible for collecting data but both will be blinded to treatment assignment. Most importantly, PRIOR to analysis, the lab manager or his associates who are not involved with the current study will de-identified key baseline and outcome data and send these to the PI, RC and steering committee for analyses.

10.3 Quality Assurance

10.3.1 Training

All research team members will perform the responsibilities as outlined by the delegation of authority log. Human Subjects Training and HIPAA training will occur as required by Stanford Policy. Additionally, all team members will receive a copy of this document, the Lab Policy Manual, and will be trained directly by PI Kong on the purpose of the project and their responsibilities. Research staff will be trained on Informed Consent, Phone Screening, and Case Report Form Completion by the PI and other senior lab research staff. The latter will monitor appropriate conduct and form completion on an ongoing basis, in collaboration with the lab manager (to be hired soon) and the PI (Kong). Research team meetings will occur frequently with the PI to ensure ongoing understanding by project staff and to address any concerns.

Regulatory and Ethical Training Requirements

The lab manager and the IRB manager will ensure that all research personnel have completed required federal and institutional training, and will maintain documentation of such. The managers will schedule annual training workshops, development of training agendas, and preparation of materials to reinforce ethical research and Good Clinical Practices. Outside speakers, such as those from the IRB, Stanford Bioethics Consultation Service, and Spectrum will be invited for training workshops.

- Human Subjects Protections: Training will include guidelines related to: HIPAA regulations and participant confidentiality, ethical research conduct, and human subject interactions.
- Conflict of Interest: Compliance with Stanford's conflict of interest policies and ongoing monitoring will also be maintained by the managers and the PI.

10.3.2 Quality Control Committee

All consent forms and CRF's will be monitored by both the research and the IRB manager. Monitoring results will be provided to the DSMB and the research team at quarterly research meetings.

10.3.3 Metrics

The metric for the primary outcome measure is self-reported pain. Daily Android data and participant questionnaires will be reviewed by the research team on a regular basis, minimally at the Baseline and Post-treatment Visits and at least once during the treatment course. The managers and the PI will also perform quality assurance by reviewing these materials on a quarterly basis.

10.3.4 Protocol Deviations

A major protocol deviation or violation includes any procedure that differs from the IRB approved protocol that was intended to eliminate an immediate hazard to the participant, was harmful, or is possible serious or continued non-compliance by a research team member.

Major protocol deviations will be communicated to the PI immediately. All events will be communicated to NCCIH within five days of the PI learning of the event. A description of the event will be included. The IRB manager will also submit this information to the IRB.

All minor protocol deviations (those that do not meet the definition of a major deviation and do not affect the interpretation or outcome of the project) will be reported to NCCIH and Stanford IRB annually.

For each participant, a Protocol Deviations Log will be maintained in the participant's data binder. A comprehensive Protocol Deviations Log will be maintained by the research assistant.

10.3.5 Monitoring

Trial Monitoring

Adverse Events, Serious Adverse Events, Unanticipated Problems, and Protocol Deviations will be monitored on a continual basis and reported per information in the relevant sections. Specifically, QA monitoring will occur once a month at the first Monday of the month, by the research team who will complete a QA monitoring CRF on REDCAP for each participant. Documents/CRF QA's will include: consent forms, patient surveys, research team CRF's, and acupuncturist's CRF's and audios. All AE's, SAE's, UP's, and PD's will be logged in separate Excel Spreadsheet for study participants. To ensure that we do not miss any items, a QA monitoring CRF is created on REDCAP for each participant and will be filled out monthly.

Additional ongoing monitoring will occur as detailed below. Results will be recorded on a monitoring log. The PI will review and sign-off on the results and any suggested resolutions.

The consent forms of the first 10 participants will be reviewed for accuracy and completion immediately after enrollment. Subsequently, consent forms will be reviewed for completion every 3 months.

The case report forms for the first four sessions completed by *each* provider will be reviewed immediately after the visits (within one day) for completeness and accuracy. Subsequently CRFs from 15-20% of each acupuncturist's sessions will be reviewed every 4 months.

The audio recordings for the first four sessions completed by *each* provider will be reviewed immediately after the visits (within one day) for protocol adherence. Subsequently recordings from 10-20% of each acupuncturist's sessions will be reviewed every 3 months (using the Ceiling function). We will review these recordings to examine adherence to communication guidelines between the provider and the participants and to protocols for intensity of the needle stimulations.

The results of the monitoring will be reported at quarterly research meetings for the project.

Case Report Form Completion

Data discrepancies will be compared to any source data and corrections made by drawing a single line through the original value, providing the correct value, dating, initialing, and providing a reason for the

correction (corrections can be made this way in REDCap). Missing data or data discrepancies that cannot be resolved by verifying source data will be left as missing. The PI will be the primary person reviewing these data. For acupuncturist's procedure notes which are standardized, the PI may train the research assistant to review these data.

Consent Form Completion

Consent forms will be evaluated for completeness of all signatures, required initials, and dates. Any missing signatures will be obtained by mailing the participant a copy of the consent form and requesting the signature with the current date. Any missing PI signatures will be obtained and dated with the current date. Participants with missing signatures who are unable, or unwilling, to provide missing signature will be withdrawn from the project and data will not be used.

11. PARTICIPANT RIGHTS AND CONFIDENTIALITY

The investigator(s)/institution(s) will permit trial-related monitoring, audits, IRB review, and regulatory inspection(s) by providing direct access to source data/documents.

11.1 Institutional Review Board (IRB) Review

This protocol and the informed consent document and any subsequent modifications will be reviewed and approved by the Stanford IRB and NCCIH.

11.2 Informed Consent Forms

A signed consent form will be obtained from each participant. Participants must be able to understand all the project procedures in order to be successfully consented. Therefore, only English-speakers will be consented for the project. Individuals under the age of 21 will not be included in the project. The consent form will describe the purpose of the project, the procedures to be followed, and the risks and benefits of participation. A signed copy will be given to each participant and this fact will be documented in the participant's record.

11.3 Participant Confidentiality

Any data, specimens, forms, reports, video recordings, and other records that leave the site will be identified only by a participant identification number (Participant ID, PID) to maintain confidentiality. All records will be kept in a locked file cabinet. All computer entry and networking programs will be done using PIDs only. Information will not be released without written permission of the participant, except as necessary for monitoring by IRB, the FDA, the NCCIH, and the OHRP.

11.4 Project Discontinuation

The project may be discontinued at any time by the IRB, the NCCIH, the OHRP, the FDA, or other government agencies as part of their duties to ensure that research participants are protected.

12. COMMITTEES

12.1 The research team

The research team will be consisted of the following:

- The PI (JK): responsible for the overall design and execution of the study, monitoring completion of CRFS by participants, research assistant and the acupuncturists. Will also serve as a backup to the research assistant for conducting the screening, baseline and post-treatment visits.
- The Research coordinator (BM): responsible for execution of the study, particularly with recruitment, conducting the screening, baseline and post-treatment visits; monitoring questionnaire completion by the participants and the acupuncturists, telephone reminders for follow up questionnaire completion by the participant.
- Acupuncturists: provide study interventions to participants, fill our CRF's, administer surveys (expectation, Vincent, blinding assessment) to participant.
- The randomizer (DB): randomize patient to Sun (verum) or Moon (sham) treatment and inform acupuncturist about assignment vial secure email.
- Project admin (CD): overseeing budget, purchasing, equipment, and facilitate communication between research team and acupuncturists.
- Pain Clinic MA (MP): help with recruitment by providing study info to patients referred to acupuncture for back pain but were denied access by their insurance. She will obtain "ok to contact" from patient and put him/her on a list and provide the study flyer to this patient.

The entire research team, particularly the PI, the acupuncturists and the RC will monitor for adverse event and protocol compliance and meet regularly (at least quarterly) to confirm safety and compliance and make reports to NCCIH or the IRB if necessary.

12.2 The supervisory committee

The supervisory committee will be consisted of the K23 mentoring team which is headed by Dr. Sean Mackey.

- Sean Mackey, MD/PhD: Dr. Mackey will oversee the timely progression of the project: that it is meeting the appropriate recruitment goals, with acceptable levels of participant retention throughout the project, and that data is collected and analyzed in a safe, blinded manner. Also he will provide critical input on manuscript preparation related to the project and development of an R01 level proposal by year 3 of the project, based on the current study.

- Margaret Chesney, PhD: Dr. Chesney will oversee the appropriate inclusion, use, and analyses of the psychological instruments used in this study; and provide mentorship to Dr. Kong on the use and interpretation of these instrument in clinical trials involving acupuncture.

- Richard Harris, PhD: Dr. Harris will over the proper inclusion, use and interpretation of the QST modalities used in this project, and mentor Dr. Kong on the overall utility of involving QST in personalized medicine involving acupuncture.

- Richard Olshen, PhD and Lu Tian PhD: The success of the project critically hinges upon the PI's ability to recruit adequate patients for each arm, manage data effectively, and analyze data via advanced statistical modeling. Drs. Olshen and Tian will provide close guidance to the PI and the research team to ensure all the above are achieved. They will also supervise the PI's training course in R-programming, machine learning and statistical modeling.

All 5 mentors will receive progress report from the PI and provide feedback on the current study and the PI's development in QST, TCM clinical trials and interpretation of psychological determinants in clinical trials involving acupuncture. The mentors will meet annually via Bluejeans and will together ensure the

proposed study is completed successfully within the proposed timeline and the PI is ready for her R-level propose by the end of year-3.

13. PUBLICATION OF RESEARCH FINDINGS

Publication of the results of this trial will be governed by the policies and procedures developed by the Executive Committee. Any presentation, abstract, or manuscript will be made available for review by the sponsor and the NCCIH prior to submission.

14. REFERENCES

1. Billman GE. Heart rate variability - a historical perspective. *Frontiers in physiology.* 2011;2:86.
2. Noda Y, Izuno T, Tsuchiya Y, et al. Acupuncture-induced changes of vagal function in patients with depression: A preliminary sham-controlled study with press needles. *Complementary therapies in clinical practice.* 2015;21(3):193-200.
3. Sparrow K, Golianu B. Does Acupuncture Reduce Stress Over Time? A Clinical Heart Rate Variability Study in Hypertensive Patients. *Medical acupuncture.* 2014;26(5):286-294.
4. Villas-Boas JD, Dias DP, Trigo PI, Almeida NA, de Almeida FQ, de Medeiros MA. Acupuncture Affects Autonomic and Endocrine but Not Behavioural Responses Induced by Startle in Horses. *Evidence-based complementary and alternative medicine : eCAM.* 2015;2015:219579.
5. Goncalves BS, Cavalcanti PR, Tavares GR, Campos TF, Araujo JF. Nonparametric methods in actigraphy: An update. *Sleep Science (Sao Paulo, Brazil).* 2014;7(3):158-164.
6. Pizzo PA, Clark NM. Alleviating suffering 101--pain relief in the United States. *The New England journal of medicine.* 2012;366(3):197-199.
7. Vickers AJ, Cronin AM, Maschino AC, et al. Acupuncture for chronic pain: individual patient data meta-analysis. *Archives of internal medicine.* 2012;172(19):1444-1453.
8. MacPherson H, Maschino AC, Lewith G, Foster NE, Witt CM, Vickers AJ. Characteristics of acupuncture treatment associated with outcome: an individual patient meta-analysis of 17,922 patients with chronic pain in randomised controlled trials. *PloS one.* 2013;8(10):e77438.
9. Sherman KJ, Cherkin DC, Ichikawa L, et al. Characteristics of patients with chronic back pain who benefit from acupuncture. *BMC musculoskeletal disorders.* 2009;10:114.
10. Sherman KJ, Cherkin DC, Ichikawa L, et al. Treatment expectations and preferences as predictors of outcome of acupuncture for chronic back pain. *Spine.* 2010;35(15):1471-1477.
11. Weidenhammer W, Linde K, Streng A, Hoppe A, Melchart D. Acupuncture for chronic low back pain in routine care: a multicenter observational study. *The Clinical journal of pain.* 2007;23(2):128-135.
12. Witt CM, Martins F, Willich SN, Schutzler L. Can I help you? Physicians' expectations as predictor for treatment outcome. *European journal of pain (London, England).* 2012;16(10):1455-1466.
13. Witt CM, Schutzler L, Ludtke R, Wegscheider K, Willich SN. Patient characteristics and variation in treatment outcomes: which patients benefit most from acupuncture for chronic pain? *The Clinical journal of pain.* 2011;27(6):550-555.
14. Yang CP, Chang MH, Li TC, Hsieh CL, Hwang KL, Chang HH. Predicting prognostic factors in a randomized controlled trial of acupuncture versus topiramate treatment in patients with chronic migraine. *The Clinical journal of pain.* 2013;29(11):982-987.
15. Linde K, Witt CM, Streng A, et al. The impact of patient expectations on outcomes in four randomized controlled trials of acupuncture in patients with chronic pain. *Pain.* 2007;128(3):264-271.

16. Wasan AD, Kong J, Pham LD, Kaptchuk TJ, Edwards R, Gollub RL. The impact of placebo, psychopathology, and expectations on the response to acupuncture needling in patients with chronic low back pain. *The journal of pain : official journal of the American Pain Society.* 2010;11(6):555-563.
17. Kong JT, Schnyer RN, Johnson KA, Mackey S. Understanding central mechanisms of acupuncture analgesia using dynamic quantitative sensory testing: a review. *Evidence-based complementary and alternative medicine : eCAM.* 2013;2013:187182.
18. Phillips K, Clauw DJ. Central pain mechanisms in chronic pain states--maybe it is all in their head. *Best practice & research. Clinical rheumatology.* 2011;25(2):141-154.
19. Woolf CJ. Central sensitization: implications for the diagnosis and treatment of pain. *Pain.* 2011;152(3 Suppl):S2-15.
20. Granovsky Y. Conditioned pain modulation: a predictor for development and treatment of neuropathic pain. *Current pain and headache reports.* 2013;17(9):361.
21. Backonja MM, Attal N, Baron R, et al. Value of quantitative sensory testing in neurological and pain disorders: NeuPSIG consensus. *Pain.* 2013;154(9):1807-1819.
22. Pincus T, McCracken LM. Psychological factors and treatment opportunities in low back pain. *Best practice & research. Clinical rheumatology.* 2013;27(5):625-635.
23. Harte SE, Clauw DJ, Napadow V, Harris RE. Pressure Pain Sensitivity and Insular Combined Glutamate and Glutamine (Glx) Are Associated with Subsequent Clinical Response to Sham But Not Traditional Acupuncture in Patients Who Have Chronic Pain. *Medical acupuncture.* 2013;25(2):154-160.
24. Yarnitsky D, Granot M, Nahman-Averbuch H, Khamaisi M, Granovsky Y. Conditioned pain modulation predicts duloxetine efficacy in painful diabetic neuropathy. *Pain.* 2012;153(6):1193-1198.
25. Bishop FL, Yardley L, Prescott P, Cooper C, Little P, Lewith GT. Psychological covariates of longitudinal changes in back-related disability in patients undergoing acupuncture. *The Clinical journal of pain.* 2015;31(3):254-264.
26. Ghoname EA, Craig WF, White PF, et al. Percutaneous electrical nerve stimulation for low back pain: a randomized crossover study. *Jama.* 1999;281(9):818-823.

15. SUPPLEMENTS/APPENDICES

Appendix 1 – Project Flow Chart

Appendix 2 – QST Instruction Manual

Appendix 3 – Acupuncture Provider's Manual
