# Supplementary material for: Effect of Electroacupuncture vs Sham Treatment on Change in Pain Severity Among Adults With Chronic Low Back Pain: A Randomized Clinical Trial
Source: JAMA Netw Open. 2020 Oct 27;3(10):e2022787. doi: 10.1001/jamanetworkopen.2020.22787 (PMC7592030; doi:10.1001/jamanetworkopen.2020.22787)
Supplement: Supplement 2. — eAppendix 1. Full Inclusion and Exclusion Criteria eTable 1. Participant Baseline Characteristics eTable 2. Blinding Analysis eTable 3. Univariate Analysis Within the Sham Arm eTable 4. Treatment Heterogeneity Analysis eAppendix 2. Sensitivity Analysis eTable 5. Summary on Type and Frequency of Adverse Events [file jamanetwopen-e2022787-s002.pdf]

Supplemental Online Content

Kong JT, Puetz C, Tian L, et al. Effect of electroacupuncture vs sham treatment on change in pain severity among adults with chronic low back pain: a randomized clinical trial. *JAMA Netw Open*. 2020;3(10):e2022787. doi:10.1001/jamanetworkopen.2020.22787

eAppendix 1. Full Inclusion and Exclusion Criteria

eTable 1. Participant Baseline Characteristics

eTable 2. Blinding Analysis

eTable 3. Univariate Analysis Within the Sham Arm

eTable 4. Treatment Heterogeneity Analysis

eAppendix 2. Sensitivity Analysis

eTable 5. Summary on Type and Frequency of Adverse Events

This supplemental material has been provided by the authors to give readers additional information about their work.

© 2020 Kong JT et al. *JAMA Network Open.*

eAppendix 1. Full Inclusion and Exclusion Criteria

Inclusion Criteria

Participants must meet all of the following inclusion criteria in order to be enrolled in the project.

- Males and females, ages 21-65
- English fluency

For back pain patients:

- Chronic Low Back Pain (cLBP)—criteria from the NIH Task Force for cLBP
    - Chronicity: cLBP "defined as a back pain problem that has persisted at least 3 months and has resulted in pain on at least half the days in the past 6 months."
    - Location: "between the lower posterior margin of the rib cage and the horizontal gluteal fold."
- Average pain over the last month ≥ 4/10 for initial eligibility
- Average pain after pre-baseline daily tracking ≥ 3.0/10

Exclusion Criteria

All candidates meeting any of the exclusion criteria at screening will be excluded from the project.

- MRI contraindications (e.g. metal implants, metallic fragments, claustrophobia)
- Ongoing legal or disability claim, Worker's Comp (permanent and stationary disability not exclusionary)
- Currently pregnant or planning to become pregnant
- Medical conditions that would interfere with study procedures, at the discretion of the study team (e.g. Heart Disease or pacemaker, active infection, current cancer diagnosis).
- Neurologic disorder, history of seizures, stroke, or brain abnormalities, which would interfere with brain integrity, at the discretion of the study team.
- Mental health conditions or treatment for mental health problems that would interfere with study procedures, at the discretion of the study team.
- Any radicular symptoms. Any identified spinal nerve root or spinal cord disease contributing to back pain.Co-morbid pain syndrome (secondary pain complaint not exclusionary if less severe)
- Opioids ≥60mg morphine equivalent units/day, benzodiazepines, beta-blockers, some antipsychotics, diabetic medications, or other medications that may interfere with study procedures at the discretion of the study team (SSRIs, anticonvulsants & thyroid medications NOT exclusionary, but TCA's and SNRI's will be excluded).
- Blood thinners (e.g. Coumadin, Plavix) at the discretion of the study team.
- Disorders indicated by the MINI 7.0 assessment including:
    - Major Depressive Episode- Current (2 weeks)
    - Major Depressive Disorder- Current (2 weeks)

© 2020 Kong JT et al. *JAMA Network Open.*

    - Suicidality- Current (Past Month)
    - Manic Episode- Current
    - Hypomanic Episode- Current
    - PTSD- Current (Past Month)
    - Alcohol Use Disorder-Past 12 Months
    - Substance Use Disorder-Past 12 Months
    - Psychotic Disorders- Lifetime
    - Mood Disorder with Psychotic Features- Current
    - Anorexia Nervosa- Current (Past 3 months)
    - Bulimia Nervosa- Current (Past 3 months)
    - Antisocial Personality Disorder- Lifetime
- Bleeding disorders at the discretion of the study team.
- Previous acupuncture treatment in the past 5 years.

© 2020 Kong JT et al. *JAMA Network Open.*

eTable 1. Participant baseline characteristics

**Baseline Variables**	**Sham Arm**	**Verum Arm**	**p between arms**
**n**	62	59	
**Age** (mean (SD))	45.58 (12.76)	45.76 (11.88)	0.935
**IsFemale = 0/1** (%)	29/33 (46.8/53.2)	23/36 (39.0/61.0)	0.495
**IsWhite = 0/1** (%)	27/35 (43.5/56.5)	22/37 (37.3/62.7)	0.606
**painT_pre** (mean (SD))	51.34 (4.85)	50.81 (3.47)	0.506
**rmdq_pre** (mean (SD))	10.03 (5.45)	10.16 (4.76)	0.894
**WSP** (mean (SD))	8.28 (9.59)	5.97 (4.20)	0.092
**mean_pptr** (mean (SD))	4.96 (2.31)	4.92 (2.18)	0.917
**mean_ts** (mean (SD))	32.34 (19.45)	31.62 (19.97)	0.842
**TS_base_T** (mean (SD))	40.88 (2.88)	40.64 (2.53)	0.628
**TS_peak_T** (mean (SD))	49.99 (2.00)	49.99 (1.43)	0.999
**CPM** (mean (SD))	-0.27 (21.68)	-3.77 (20.01)	0.37
**sets_pos** (mean (SD))	10.81 (2.90)	10.98 (2.38)	0.737
**sets_neg** (mean (SD))	9.50 (2.55)	9.13 (2.86)	0.475
**pcs_sum** (mean (SD))	14.37 (9.81)	14.86 (9.25)	0.775
**pseq_sum** (mean (SD))	293.39 (83.61)	303.73 (87.71)	0.508
**csq** (mean (SD))	71.29 (18.32)	74.44 (18.46)	0.348

© 2020 Kong JT et al. *JAMA Network Open.*

eTable 2. Blinding Analysis.

2a. Raw data.

Tx Assigned	guessed real	guessed sham	don't know	total
real	28	4	16	48
sham	11	20	15	40

2b. Bang's Blinding Index and 95% CI for Real and Sham Arms.

	Blinding Index	SD	confidence	95% CI	
Real Arm	0.5	0.645497	0.182609	0.31739	0.68261
Sham Arm	0.2	0.851102	0.263754	−0.0681	0.45941

2c. Interpretation.

- Real arm: 50% of participants correctly guessed the treatment identity beyond chance.
- Sham arm: 20% of participants correctly guessed the treatment identity beyond chance.

© 2020 Kong JT et al. *JAMA Network Open.*

eTable 3. Section 4. Univariate analysis within the sham arm.

Baseline Predictors	outcome = **Δpain**		outcome = **ΔRMDQ**	
	r (partial correlation)	***p***	***r*** (partial correlation)	***p***
Age	0.071	0.617	0.110	0.436
csq	0.001	0.995	-0.185	0.189
pseq_sum	-0.059	0.677	-0.164	0.244
pcs_sum	-0.207	0.140	0.182	0.197
mean_pptr	0.031	0.825	0.176	0.212
TS_base_T	0.116	0.412	0.143	0.313
TS_peak_T	0.052	0.713	0.130	0.360
mean_ts	-0.008	0.955	0.003	0.982
CPM	0.041	0.784	0.021	0.889
sum (wsp)	0.172	0.229	0.099	0.489
sets_pos	-0.148	0.301	-0.146	0.306
sets_neg	-0.266	0.059	-0.200	0.160
IsFemale	-0.31	0.81	0.15	0.9
IsWhite	0.12	0.93	0.03	0.98

Footnote:

1. No statistically significant correlations are observed.

2. Since the outlier was randomized to the verum arm, the partial correlations within the sham arm is unaffected by the outlier.

© 2020 Kong JT et al. *JAMA Network Open.*

eTable 4. Section 5: Treatment Heterogeneity Analysis

Model: $Y = Y_0 + \beta_T$*Treatment + β_P*Predictor + $\beta_{interaction}$*Predictor*Treatment

	Outcome = ΔPain			Outcome = ΔRMDQ	
**Predictor Variable**	**$\beta_{interaction}$**	**p-value**		**$\beta_{interaction}$**	**p-value**
**base-line pain or RMDQ**	-0.27	0.35		-0.19	0.24
**Age**	-0.1	0.30		0.02	0.79
**IsFemale**	4.25	0.051.		1.11	0.51
**IsWhite**	4.10	0.06.		2.83	0.09
**sets_pos**	-0.44	0.19		0.10	0.71
**sets_neg**	0.59	0.06.		0.33	0.85
**wsp (sum)**	-0.11	0.57		-0.31	0.7
**csq**	-0.09	0.12		-0.02	0.68
**pseq_sum**	0.003	0.82		0.01	0.26
**pcs_sum**	0.26	***0.03***		-0.11	0.22
**mean_pptr**	-0.46	0.33		-0.36	0.32
**mean_ts**	0.11	0.07.		0.03	0.56
**TS_base_T**	0.54	0.20		0.05	0.87
**TS_peak_T**	0.45	0.55		0.15	0.79
**CPM**	-.001	0.98		0.04	0.40

Footnote:

1. Even though pcs_sum had p=0.03, it was NOT statistically significant after accounting for multiple comparisons.

2. Highlight signifies interactions with the lowest p-value, and to be included in the multi-variable prediction model in section 5 of Results in the main manuscript.

© 2020 Kong JT et al. *JAMA Network Open.*

eAppendix 2. Sensitivity analysis:

A: treatment effect analysis – histogram and bloxplot of pain reduction reveals a single outlier with high increase in pain. (The single outlier is highlighted in RED).

Histogram of Δpain in full sample

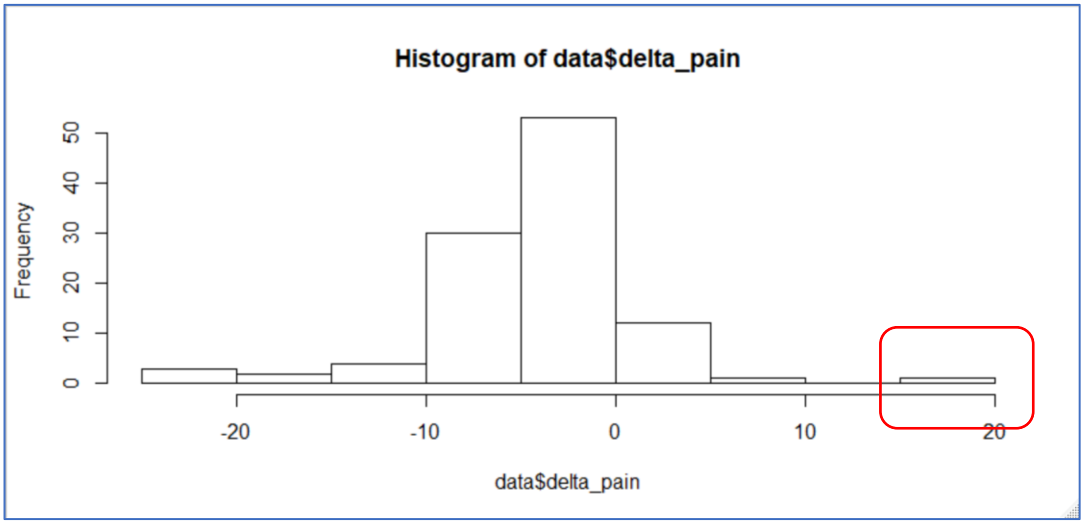

Boxplot of Δpain in verum arm

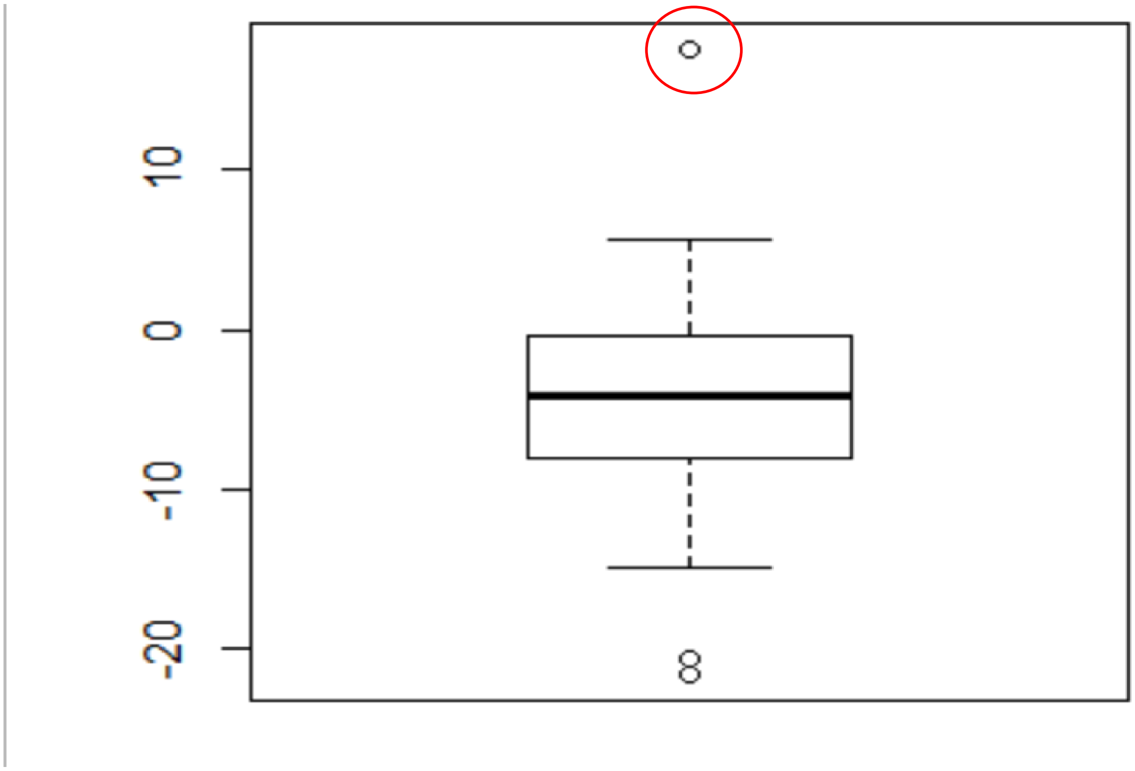

© 2020 Kong JT et al. *JAMA Network Open.*

B: predictive relationships with or without the outlier within verum arm.

Legend: The predictive relationship between positive expectations (and temporal summation) and pain reduction disappeared after removing the outlier (2A and 2B) while the relationship between coping and RMDQ reduction remained unchanged after removing the outlier (2C).

6a. Partial correlation between Δpain (residual) and positive expectations in Verum Arm, with or without the outlier.

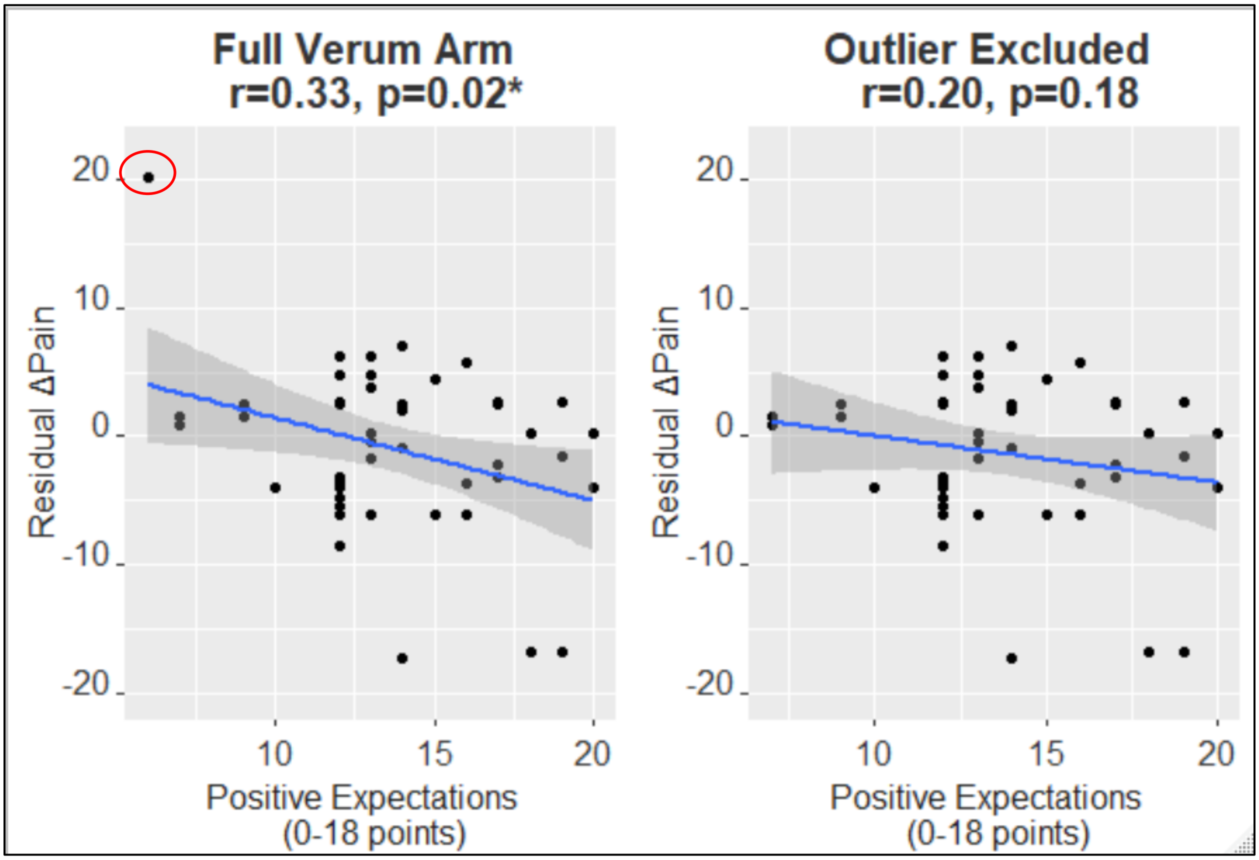

© 2020 Kong JT et al. *JAMA Network Open.*

6b. Partial correlation between Δpain (residual) and temporal summation (mean_TS) in Verum Arm, with or without the outlier.

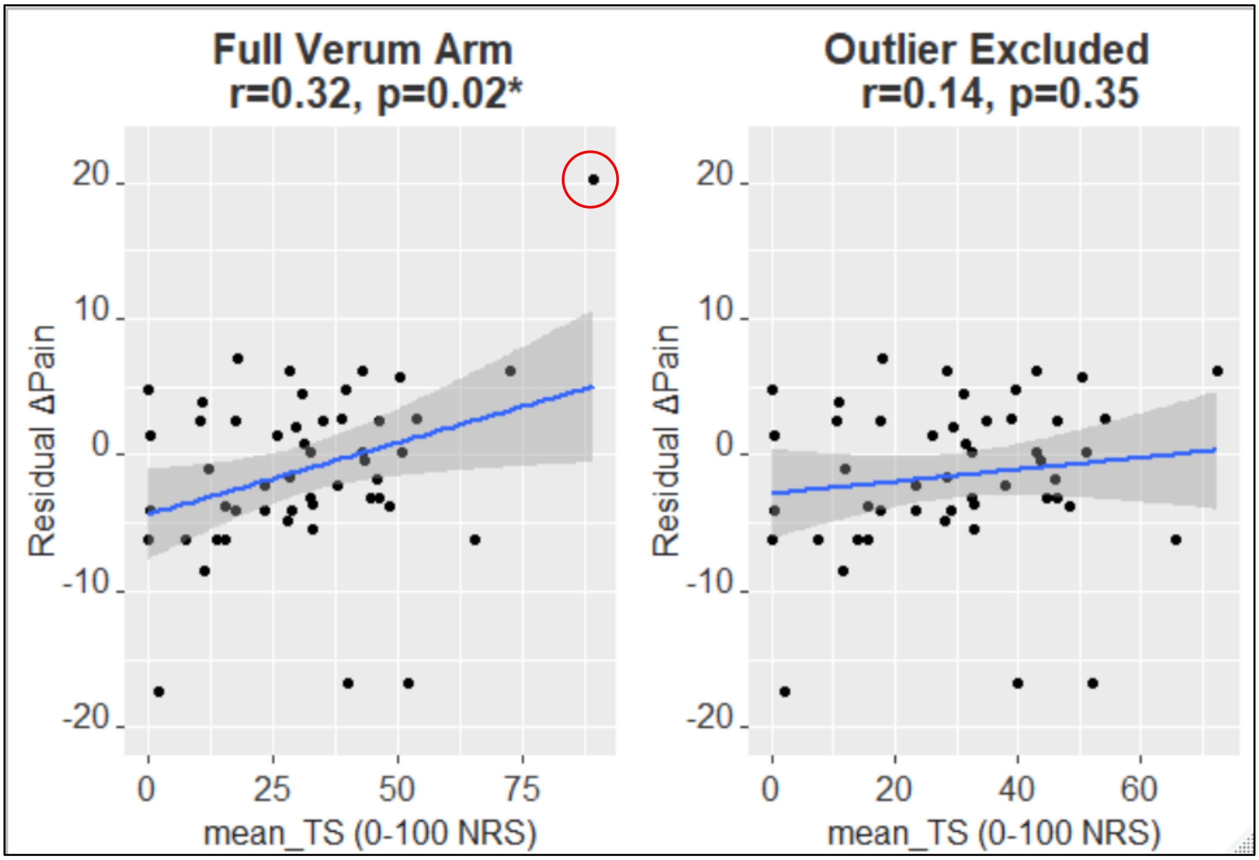

© 2020 Kong JT et al. *JAMA Network Open.*

6c. Partial correlation between ΔRMDQ (residual) and Coping Strategies (CSQ) in Verum Arm remained stable regardless of the outlier's presence.

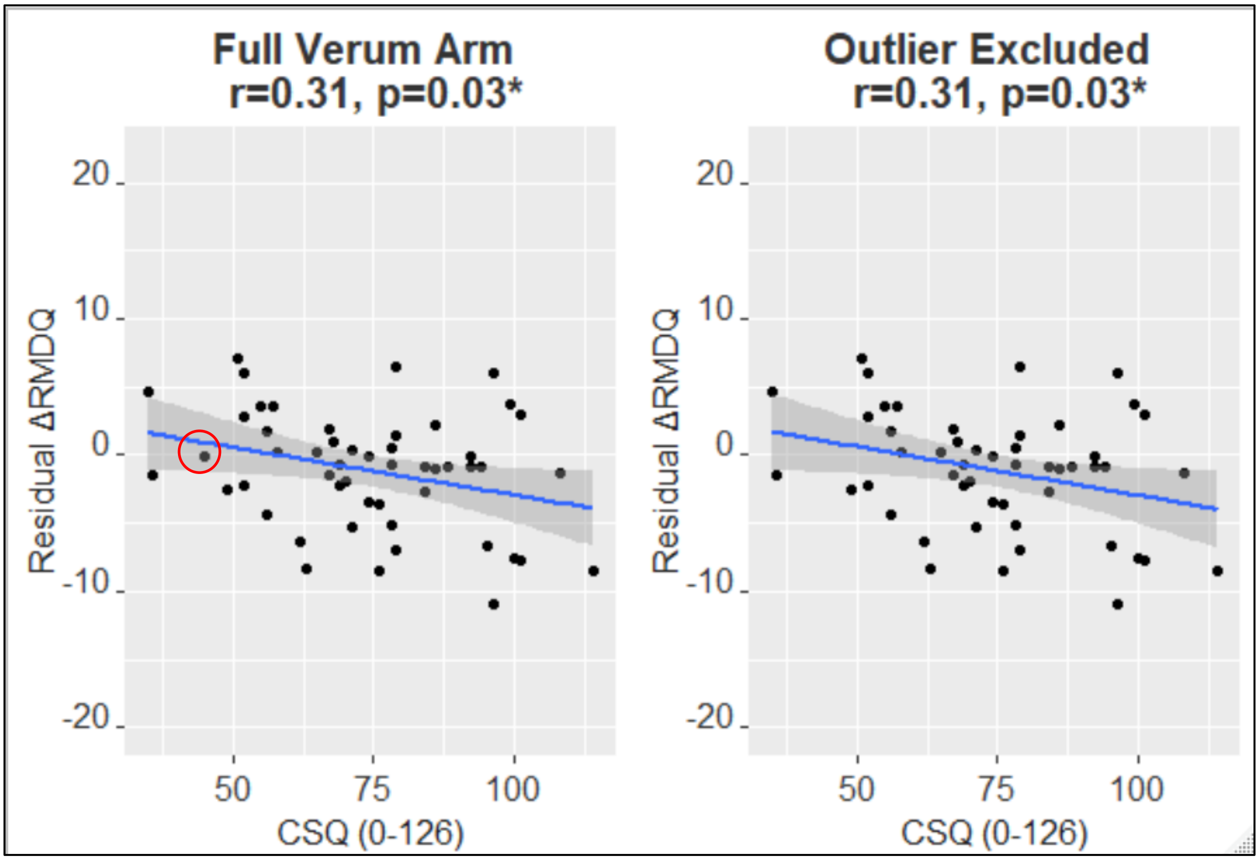

Footnote: recall we modified the CSQ total score such that it did not contain questions from the catastrophizing category.

© 2020 Kong JT et al. *JAMA Network Open.*

eTable 5. Summary on type and frequency of adverse events

Adverse Event Type	Total Number of Participants in Combined Arms	Total Number of Participants in Verum Arm	Total Number of Participants in Sham Arm
Reaction to Acupuncture Treatment *Examples: minor pain, bruising, skin rash, and slight bleeding at needle site; as well as mild reaction to prone (face-down) position including nausea, dizziness, and mild back ache.*	34 (28 %)	24 (41%)	10 (16%)
Back Pain Flairs*	11 (9 %)	5 (8%)	6 (10%)
Reaction to Assessments (pressure testing, heat testing, cold water bath) *Examples: mild soreness; bruising*	21 (17 %)	10 (17%)	11 (18%)
Other* *Examples: illness (cold, flu); injury/accident; other pain flare-ups (not in the back); reaction to change in medication; allergic reaction; migraine*	34 (28 %)	18 (31%)	16 (26%)
Footnote:			
(*): Adverse events not related to study, including the back pain flairs which participants attributed to work and recreational activities.			
Total Participants Randomized to Study	121		
Total Verum Arm Participants	59		
Total Sham Arm Participants	62		

© 2020 Kong JT et al. *JAMA Network Open.*
