# Supplementary material for: Effect of Electroacupuncture vs Sham Treatment on Change in Pain Severity Among Adults With Chronic Low Back Pain: A Randomized Clinical Trial
Source: JAMA Netw Open. 2020 Oct 27;3(10):e2022787. doi: 10.1001/jamanetworkopen.2020.22787 (PMC7592030; doi:10.1001/jamanetworkopen.2020.22787)
Supplement: Supplement 3. — Data Sharing Statement [file jamanetwopen-e2022787-s003.pdf]

Data Sharing Statement

Kong. Effect of Electroacupuncture vs Sham Treatment on Change in Pain Severity Among Adults With Chronic Low Back Pain. *JAMA Netw Open*. Published October 27, 2020.
10.1001/jamanetworkopen.2020.22787

Data
Data available: Yes
Data types: Deidentified participant data, Data dictionary
How to access data: jtkong@stanford.edu
When available: With publication

Supporting Documents
Document types: Statistical/analytic code, Informed consent form
How to access documents: jtkong@stanford.edu
When available: With publication

Additional Information
Who can access the data: researchers whose proposed use of the data has been approved
Types of analyses: Main outcome analysis
Mechanisms of data availability: after approval of a proposal, or with a signed data access agreement
